# *De novo* Design of Near-Infrared Fluorescence-activating Proteins

**DOI:** 10.1021/jacs.5c19594

**Published:** 2026-06-02

**Authors:** Yulai Liu, Bernardo A. Arús, Kanuj Mishra, Taolin Wang, Kun Zhang, Michael P. Luciano, Venu G. Bandi, Akaash Kumar, Ziyu Guo, Yun Guan, Matthew J. Bick, Miaomiao Xu, Jakob Lingg, Jessica Bae, Alex Kang, Stacey R. Gerben, Asim K. Bera, Joshua C. Vaughan, James D. Manton, Emmanuel Derivery, Martin J. Schnermann, Andre C. Stiel, Oliver T. Bruns, Chunfu Xu, David Baker

**Affiliations:** 1 Institute for Protein Design and Department of Biochemistry, University of Washington, Seattle, WA 98195, USA.; 2 German Cancer Research Center (DKFZ), Heidelberg 69120, Germany; National Center for Tumor Diseases (NCT/UCC), Dresden 01307, Germany; Medizinische Fakultät and University Hospital Carl Gustav Carus, Technische Universität Dresden, Dresden 01062, Germany; Helmholtz-Zentrum Dresden-Rossendorf (HZDR), Dresden 01328, Germany.; 3 Helmholtz Pioneer Campus, Helmholtz Zentrum München, Neuherberg 85764, Germany.; 4 Institute of Biological and Medical Imaging, Helmholtz Zentrum München, Neuherberg 85764, Germany.; 5 National Institute of Biological Sciences, Beijing 102206, China.; 6 Tsinghua Institute of Multidisciplinary Biomedical Research, Tsinghua University, Beijing 102206, China.; 7 College of Life Sciences, Beijing Normal University, Beijing 100875, China.; 8 Chemical Biology Laboratory, Center for Cancer Research, National Cancer Institute, Frederick, MD 21702, USA.; 9 MRC Laboratory of Molecular Biology, Francis Crick Avenue, Cambridge CB2 0QH, UK.; 10 Department of Chemistry, University of Washington, Seattle, WA 98195, USA.; 11 School of Geographic and Biologic Information, Nanjing University of Posts and Telecommunications, Nanjing 210023, China.; 12 Department of Neurobiology and Biophysics, University of Washington, Seattle, WA 98195, USA.; 13 Institute of Biophysics and Physical Biochemistry, Regensburg Center for Biochemistry, University of Regensburg, D-93040 Regensburg, Germany.; 14 Regensburg Center for Ultrafast Nanoscopy (RUN), University of Regensburg, D-93040 Regensburg, Germany.; 15 Howard Hughes Medical Institute, University of Washington, Seattle, WA 98195, USA.

**Keywords:** Peptide and proteins, Protein structure, Protein engineering, Imaging probes, Biological imaging

## Abstract

Protein-based fluorescence imaging is a powerful modality for visualizing diverse biological processes. Biological imaging in the near-infrared (NIR, 800–1000 nm) and shortwave infrared (SWIR, 1000–2000 nm) ranges confers a number of photophysical advantages, but remains a challenge in practice due to the dearth of suitable protein probes in these optical windows. To address this limitation, we sought to develop a general approach integrating computational protein design with organic synthesis for creating long-wavelength fluorescence-activating proteins from scratch. We used this approach to de novo design proteins that specifically bind to synthetic merocyanine dyes, forming Schiff base covalent linkages, which when protonated activate fluorescence with large redshifts in both excitation and emission wavelengths. We describe a designed far-red fluorescence-activating protein, MC7BP34, with a brightness greater than that of existing fluorescent proteins in a similar wavelength range, and an NIR design MC9BP81 with excitation at 892 nm and emission extending into the SWIR range with higher contrast and imaging sensitivity in vivo than the previously developed iRFP720 (excitation 672 nm) owing to the reduced tissue autofluorescence at longer wavelengths. Our results are a substantial step toward genetically encodable probes in the SWIR region, and our approach lays the groundwork for the development of NIR biosensors for specific biological applications.

## Graphical Abstract

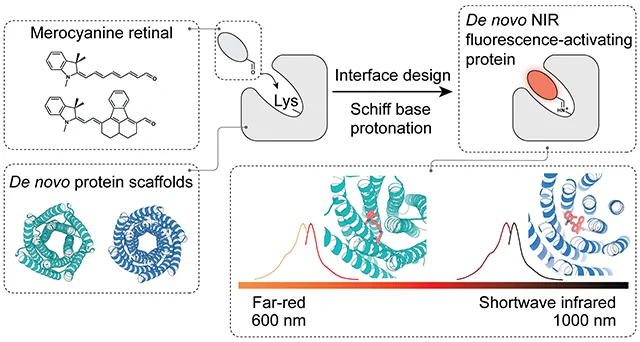

## Introduction

The engineering of natural fluorescent proteins has led to an extensive repertoire spanning the entire visible light range which enables the visualization of a wide array of functional processes in biological systems. However, there is an unmet need for fluorescent proteins suitable for shortwave infrared (SWIR) fluorescence imaging. SWIR light has reduced tissue scattering and autofluorescence, allowing it to achieve deeper penetration depth;^1–4^ in tissue phantoms, SWIR imaging has enabled penetration depths beyond 1 cm.^5^ Small molecule dyes, carbon nanotubes, and inorganic nanoparticles have been used for biological imaging in this spectral regime,^6–14^ but genetically encoded protein-based probes would offer more precise temporal and spatial control in biological systems since expression of the probe can be induced at specific times in desired cell types or tissues, thereby opening new avenues for deep tissue imaging. Different approaches have been attempted with various degrees of success including taking advantage of emission tails of fluorescent proteins at the extreme red end of the visible spectrum,^15^ solubilizing organic dyes using plasma proteins,^16^ the combination of these two approaches,^17^ redesigning natural fluorescent proteins,^18^ optimizing self-labeling protein-dye systems,^19,20^ and designing chromophore-binding proteins.^21–23^ However, the optical properties (for example, molecular brightness and bathochromic shift) of such proteins at long wavelengths, including those with intrinsic chromophores^24^ and those that bind synthetic dyes,^19–23^ are suboptimal compared to those operating in shorter wavelength ranges, driving the quest for near-infrared (NIR) protein probes with well-defined chromophore-protein interfaces suitable for SWIR imaging. The ability to design proteins with long-wavelength fluorescence from scratch could substantially enrich the repertoire of tools available for deep tissue imaging, thereby amplifying its utility in biomedical research.

**Figure 1.**
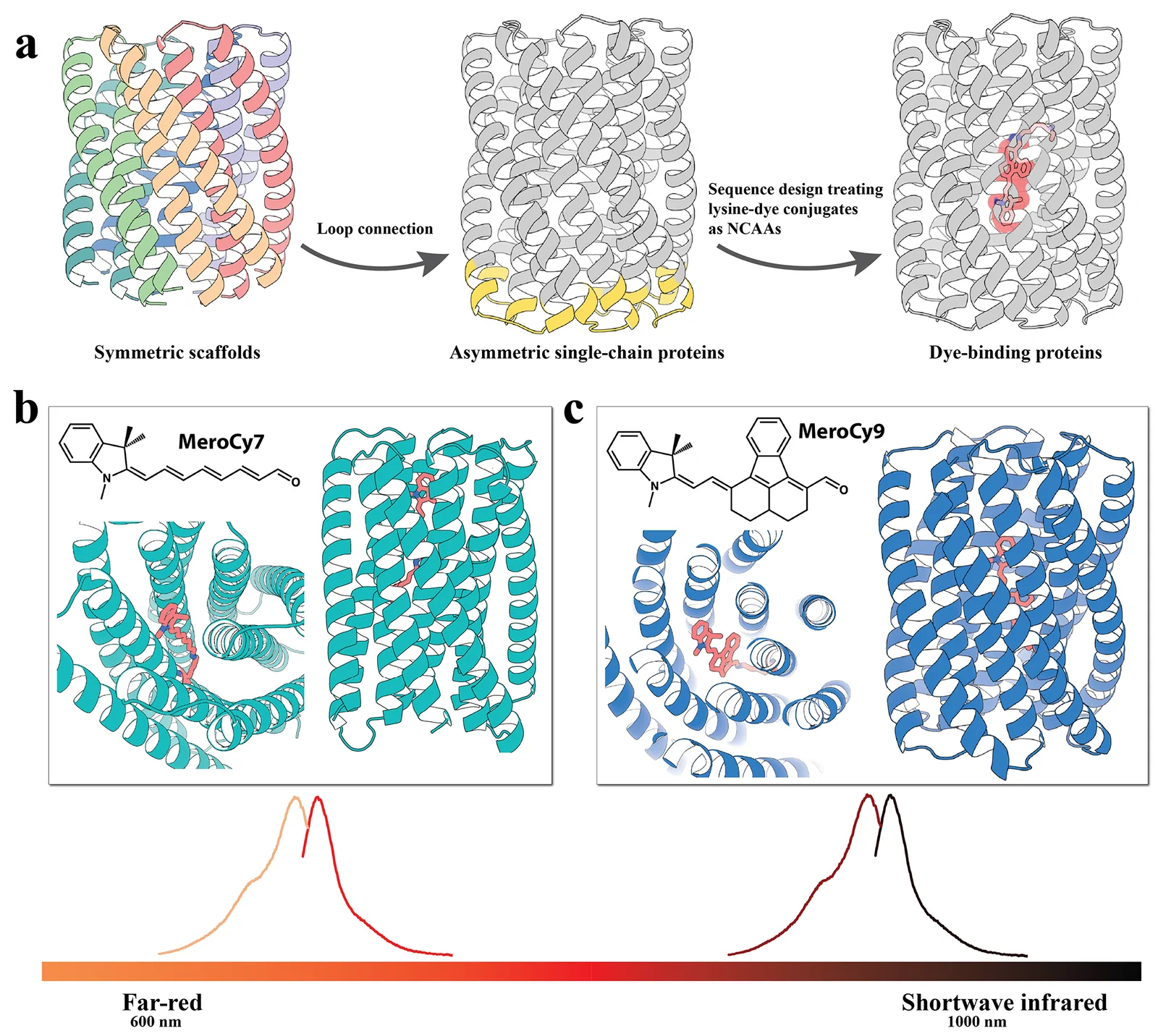
Computational design strategy. (**a**) General procedure for the design of fluorescence-activating proteins. Symmetric helical bundle oligomers with central pores suitable for ligand binding are generated using parametric backbone design and experimentally characterized. Those that are found experimentally to adopt the target structure are connected into single-chain proteins with short loops and the residues surrounding the binding pocket are optimized by RosettaDesign to bind merocyanine retinals. To enable simultaneous sampling of the ligand conformation and design of the amino acid identities of the surrounding pocket, we treat the merocyanine retinal-lysine conjugates as NCAAs in Rosetta and allow them to sample a wide range of rotameric states. (**b**) Design model of a far-red fluorescence-activating protein utilizing the MeroCy7 dye (chemical structure shown in the figure) based on a pentameric two ring helical bundle. (**c**) Design model of a fluorescence-activating protein predicted to emit in the SWIR range utilizing the bulkier MeroCy9 dye and based on a hexameric two ring helical bundle. Spectra are for illustrative purposes only.

We set out to develop a broadly applicable approach for the design of fluorescence-activating proteins with tunable spectral properties across a wide wavelength range by combining the strengths of computational protein design and organic synthesis. Our approach has two steps. First, we identify and synthesize fluorogenic small molecules that become fluorescent in the desired wavelength range after (but not before) conjugation to a protein. Second, we *de novo* design proteins with chemically and shape complementary pockets for binding the chosen molecules, and with a suitably placed amino acid side chain nucleophile for covalent conjugation. We chose merocyanine retinals as the dye molecules because of two advantageous properties:^25–28^ first, the red-shifted long-wavelength emission of these molecules is dependent on both the formation of a Schiff base linkage with an activated lysine residue and a crucial subsequent protonation of the imine group, which depends sensitively on and hence can be tuned by the protein environment, and second, the spectra of the resulting fluorescent protein-dye complex can be adjusted by modifying polyene moiety present in merocyanine retinals. We focused on MeroCy7 and MeroCy9 (Figure 1b and c) which upon binding to proteins, have the potential to emit in the NIR-SWIR range.

## Results

### Design of a Far-Red Fluorescence-Activating Protein

On the protein side, we began with the design of oligomeric helical proteins featuring a central pore that has the correct overall shape to bind elongated small molecules. Using parametric protein design,^29^ we designed and experimentally verified two distinct oligomeric proteins: a pentameric and a hexameric structure, both comprising concentric rings of α-helices. The hexameric protein (WSHC6, PDB: 6TJ1) has been previously reported^30^. The pentameric protein (denoted WSHC5, for water-soluble hairpin C5), similar to WSHC6, was well expressed in *Escherichia coli* and could be efficiently purified using nickel-affinity chromatography and size-exclusion chromatography (SEC). The circular dichroism spectrum of WSHC5 showed that the protein had the expected α-helical secondary structure and was stable to thermal denaturation up to 95 °C. The crystal structures of these proteins superimpose closely with the design models with Cα-RMSD of 0.86 Å (WSHC5, PDB: 8U5W) and 0.89 Å (WSHC6), respectively.

We next explored the design of dye-binding proteins based on these validated channel-containing oligomeric scaffolds. Recent advances in computational design have enabled the creation of small-molecule-binding proteins for diverse ligands.^31–35^ However, efficiently co-sampling ligand conformations alongside protein side chains within the binding pocket remains a key challenge. We chose an approach which bypasses the conventional docking procedure typically used to place ligands in protein pockets. Instead, we explicitly modeled the Schiff base between the merocyanine retinals and lysine residues, treating the combination as an unnatural amino acid within the Rosetta noncanonical amino acid (NCAA) framework.^36,37^ This allowed us to use the Rosetta machinery to simultaneously pack the ligand and the surrounding protein residues, eliminating the need for global docking.

**Figure 2.**
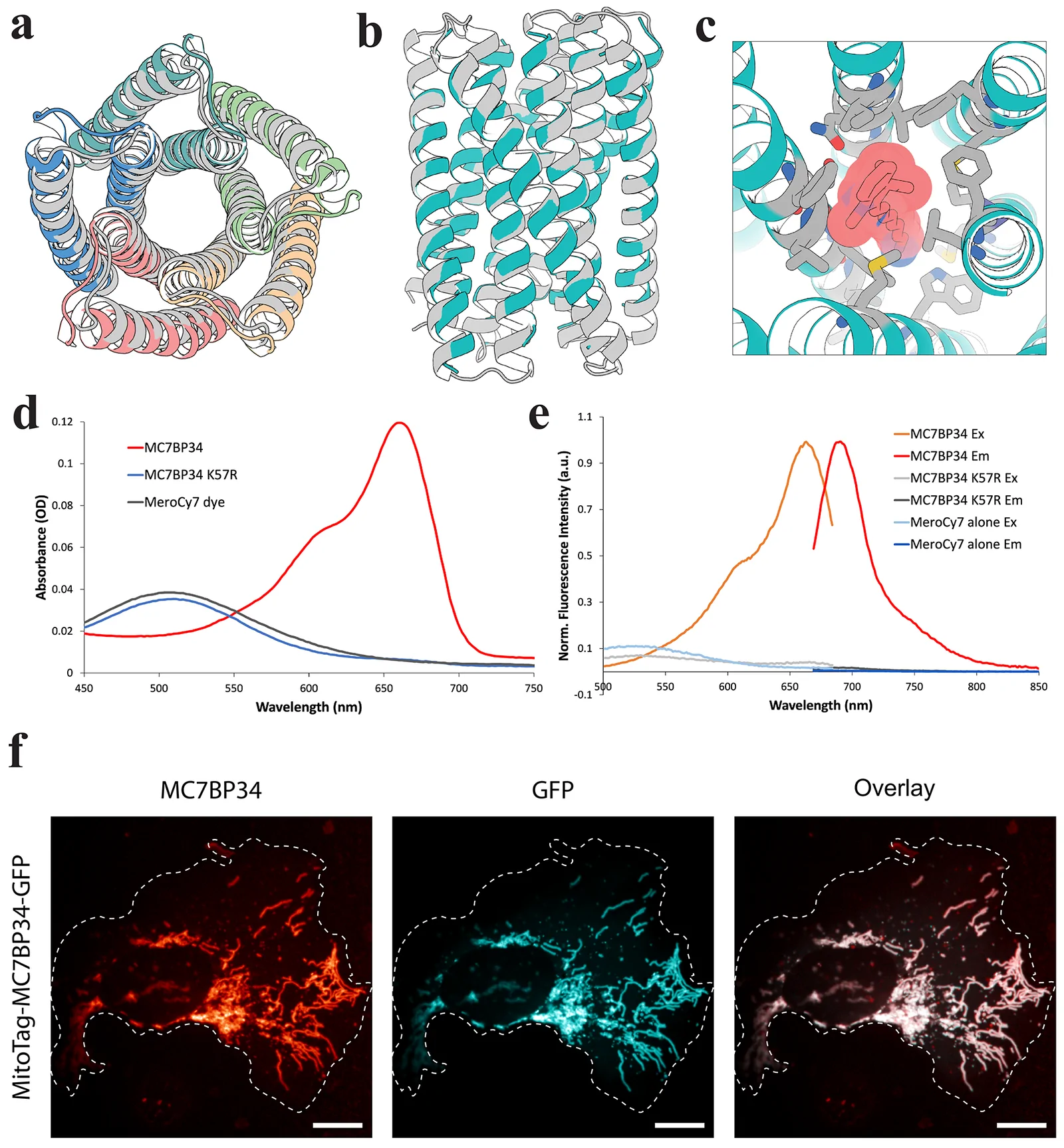
Design and experimental validation of a far-red fluorescence-activating protein. (**a**) Crystal structure (color) of the designed pentameric two ring helical bundle matches well with the design model (gray) with a Cα-RMSD of 0.86 Å. (**b**) Crystal structure (teal) of the designed far-red fluorescence-activating protein MC7BP34 aligns with the design model (gray). (**c**) Ligand binding pocket in the design model. Modeling suggests the small molecule should fit closely with the surrounding protein residues, but it is not discernible in the crystal structure due to the low resolution. (**d**) Absorption spectra of MC7BP34 and the K57R mutant incubated with MeroCy7 dye compared to MeroCy7 alone. The absorption peak shifts from 505 nm for the unbound dye to 655 nm for the protein-dye complex; the knockout mutant K57R does not induce a shift in the absorption spectra, suggesting that the dye molecule binds specifically to the lysine residue as designed. The K57R mutant remains monomeric and folded (Figure S6). (**e**) Excitation and emission spectra of MC7BP34 and the K57R mutant incubated with MeroCy7 dye, or MeroCy7 alone. (**f**) U2OS cells expressing a MC7BP34-GFP fusion targeted to mitochondria incubated with 0.3 μM MeroCy7 for 15 min then washed and imaged live by spinning disk confocal microscopy (single plane). Scale bars: 10 μm.

As a first test of the approach, we sought to design all-trans retinal binders based on the pentameric scaffold (Figure 2a) which contains a central channel with the appropriate dimensions to accommodate the elongated ligand. We introduced short loops between the pentamer subunits to create a single chain protein to enable the breaking of structural symmetry (the ligand does not have cyclic symmetry) and designed the sequence using RosettaDesign methodologies (see Supporting Information and Figure 1a), treating retinal-lysine conjugate as an NCAA. After *in silico* selection based on the shape complementarity and the interface area between all-trans retinal and the designed binding proteins, we obtained synthetic genes for 12 designs, out of which 10 exhibited successful expression in *E. coli* and bound retinal, as confirmed by UV-vis absorption spectroscopy. This high success rate likely reflects the hydrophobic nature of the merocyanine retinal, akin to the interaction of small molecules with the hydrophobic pockets in helical bundle proteins.^38^

Encouraged by this high success rate, we next sought to design binding proteins for MeroCy7 (MC7, Figure 1b), a red-shifted modified merocyanine aldehyde inspired by previous work^26^ (see Supporting Information for synthesis). Given the high similarity between all-trans retinal and MeroCy7, we started with the retinal binding design, RET2, that exhibited the highest expression and least aggregation. Using the design model as a guide, we redesigned the neighboring residues close to the Schiff base and the aromatic ring of MeroCy7, with the goal of facilitating the protonation of the Schiff base nitrogen and stabilizing the positive charge. We found that two designed glutamate substitutions of RET2 (resulting in design RET2_EE) led to MeroCy7 fluorescence activation within the far-red region (Figure S1). To further enhance the fluorescence properties, we conducted site-saturation mutagenesis (SSM) focusing on residues near the fluorophore, integrated the fluorescence-enhancing mutations identified from the SSM library screening, and redesigned the loops and positions distal to the dye binding site to optimize yield. The resulting optimized far-red design, MC7BP34, exhibits robust fluorescence emission at 685 nm with an excitation maximum at 663 nm when bound to MeroCy7 (Figure 2d–e). Intact protein Electrospray Ionization Mass Spectrometry (ESI-MS) of MC7BP34 labeled with MeroCy7 revealed a 262 Da mass increase, consistent with covalent attachment of the dye to the protein (Figure S2). We determined the crystal structure of labeled MC7BP34 (PDB: 8U1A) and found that it aligned well with our design model (Figure 2b). Due to the low resolution, the dye molecule is not discernible in the crystal structure. However, the blue color of the crystals is consistent with the large red-shift of the protein-dye complex (Figure S3) and suggests the presence of the dye covalently linked to the protein via a protonated Schiff base. We used site-directed mutagenesis to confirm the correct binding of MeroCy7 to the designed site. Fluorescence was completely abolished when the active lysine residue was mutated to arginine (Figure 2d–e), indicating that MeroCy7 binds specifically to the lysine residue in the designed pocket. Using an integrating sphere, the fluorescence quantum yield of MC7BP34 was determined to be 0.44, higher than that of Cy5 and Alexa647 dyes (0.28 and 0.33 in aqueous solution^39^, respectively) with similar excitation and emission spectra. With a molar extinction coefficient of 150,000 M^−1^ cm^−1^, MC7BP34 is brighter than all the fluorescent proteins documented in FPbase^24^ (Figure S4b) and is among the brightest synthetic chromophore-binding proteins in the far-red wavelength region^19–21^. Fluorescence activation of MeroCy7 upon mixing with MC7BP34 reached a plateau within ~30 min (Figure S5).

To explore the use of MC7BP34 as a genetically encoded far-red dye in cells, we cloned and expressed MC7BP34 flanked with a C-terminal GFP and an N-terminal mitochondrial-targeting sequence (MitoTag-MC7BP34-GFP) in U2OS cells. Upon incubation with MeroCy7, strong far-red signal could be detected on the mitochondria (Figure 2f, note that the GFP and MC7BP34 signals colocalize). MC7BP34 is well adapted for live cell imaging using a pulse-chase labeling strategy (Movie S1). Indeed, free MeroCy7 slowly accumulated in intracellular compartments (Figure S7), but since MC7BP34 covalently interacts with MeroCy7, unbound dye can simply be washed away before imaging (see Supporting Information for methods). Specific dye labeling yielded significantly higher fluorescence intensity than nonspecific intracellular accumulation of dye (Figure S8), resulting in high imaging contrast.

Self-labeling proteins such as HaloTag and SNAP-tag are widely used in microscopy imaging experiments. A key consideration in these experiments, particularly in multicolor imaging, is the orthogonality of the labeling chemistries. To evaluate this for MC7BP34, we tagged the histone H2B with MC7BP34, the outer mitochondrial membrane protein Tomm20 with HaloTag7, and the tubulin with SNAPf, respectively, and coexpressed these fusion constructs in HEK293T cells. After cells were labeled with the SNAP-Cell Oregon Green substrate, the Janelia Fluor 549 HaloTag ligand and MeroCy7, distinct green, orange and far-red signals were observed on microtubules, mitochondria, and cell nuclei, respectively (Figure S9). No cross-reactivity was detected, indicating that MC7BP34 provides an additional orthogonal channel for multicolor imaging.

### Design of an NIR Fluorescence-Activating Protein

**Figure 3:**
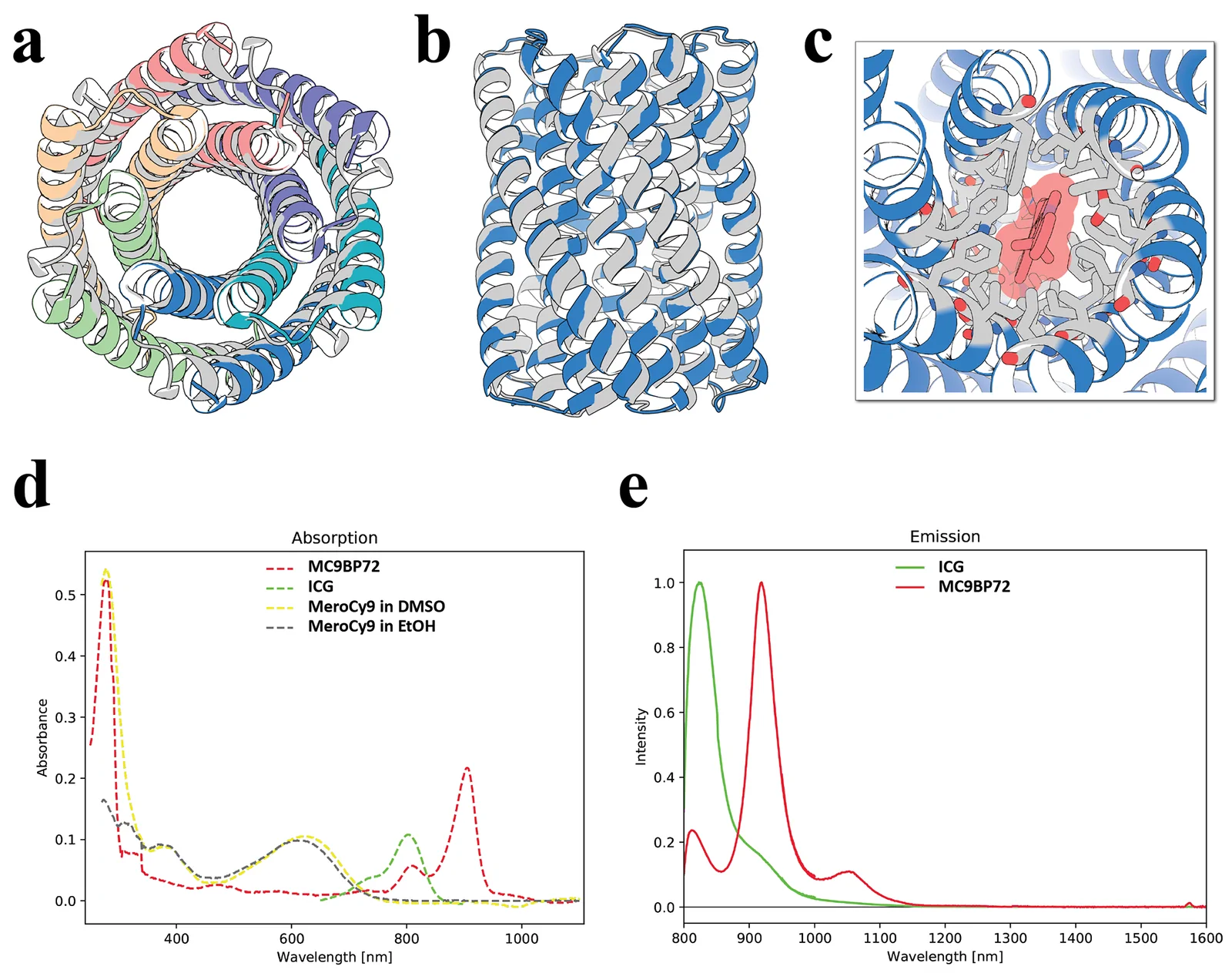
Design and experimental validation of a SWIR fluorescence-activating protein. (**a**) Superposition of the crystal structure (color) and the design model (gray) of the hexameric two ring helical scaffold used for the SWIR design. The larger internal channel of this scaffold is suitable to bind bulkier ligand compounds. (**b**) The AlphaFold predicted model (blue) of the MeroCy9 binding SWIR protein MC9BP72 aligns with the design model (gray) with a Cα-RMSD of 1.41 Å. (**c**) The ligand binding pocket in the design model is complementary in shape to the MeroCy9 dye. (**d**) Absorption spectra of free MeroCy9 dye (10 μM), MC9BP72 (10 μM), and ICG (1 μM). The protein-dye complex absorbs light at 904 nm, representing an almost 300 nm red shift from the free dye’s absorbance and a 100 nm red shift compared to ICG. **e**) Fluorescent emission spectra for ICG (green) and MC9BP72-MeroCy9 complex (red) obtained at an excitation wavelength of 785 nm. The emission peak of the designed protein-dye complex shifts over 100 nm toward the longer wavelength compared to that of ICG.

Building on our success in designing far-red fluorescence-activating proteins, we next sought to create proteins active in the NIR region by designing binders for a bulkier merocyanine retinal molecule, MeroCy9 (MC9; Figure 1c), which could fluoresce at wavelengths in the SWIR range. MeroCy9 has an additional aryl ring fused onto the chromophore similar to that in a symmetrical cyanine with SWIR absorbance/emission^12^. We synthesized MeroCy9 using a tandem Suzuki reaction from the corresponding dichloride precursor, as described in the supplement. For the binding protein design, we selected the hexameric scaffold with the larger interior pore, WSHC6 (Figure 3a), and followed the general strategy used for the MeroCy7 binder design. We connected the subunits into a single chain using short loops and used Rosetta to optimize the sequence. As with MeroCy7, we treated MeroCy9-lysine as an NCAA and only allowed it at pore-facing positions. Following design and *in silico* selection, we obtained synthetic genes and expressed the proteins in *E. coli*. Initial designs aggregated, which we hypothesized resulted from the sequence repetition arising from bridging the hexameric protein into a single chain (leading to many very similar possible inter and intra repeat interactions). To solve this problem, we redesigned the entire protein breaking the repetitive sequence pattern and used Rosetta HBNet^40^ to search for hydrogen-bonding networks to favor the desired folded state. We also positioned glutamate and aspartate residues around the small molecule to facilitate protonation of the Schiff base. Of 12 such designs, absorption spectroscopy indicated that two bound the dye. We carried out a second round of design focusing on redesigning the core of the most promising candidate to further improve the binding and enhance the extinction coefficient. This led to design MC9BP72 which binds MeroCy9 and absorbs light at 904 nm (Figure 3d), an almost 300 nm red shift compared to the absorbance of the free dye. The predicted structure of this protein using AlphaFold^41^ closely matches the design model (Figure 3b). Site-directed mutagenesis experiments confirmed interaction of MeroCy9 to the intended lysine residue in the designed pocket (Figure 3c): the absorption peak disappeared upon mutating the active lysine to arginine (Figure S1). Following excitation of the MC9BP72-MeroCy9 holo complex at 785 nm, fluorescence was observed by SWIR spectroscopy at 920 nm (Figure 3e), a red shift of more than 100 nm compared to indocyanine green^42^ (ICG, 810 nm emission), a dye commonly used in clinical imaging. The complex also has a secondary emission peak at 1050 nm which extends beyond 1100 nm.

**Figure 4:**
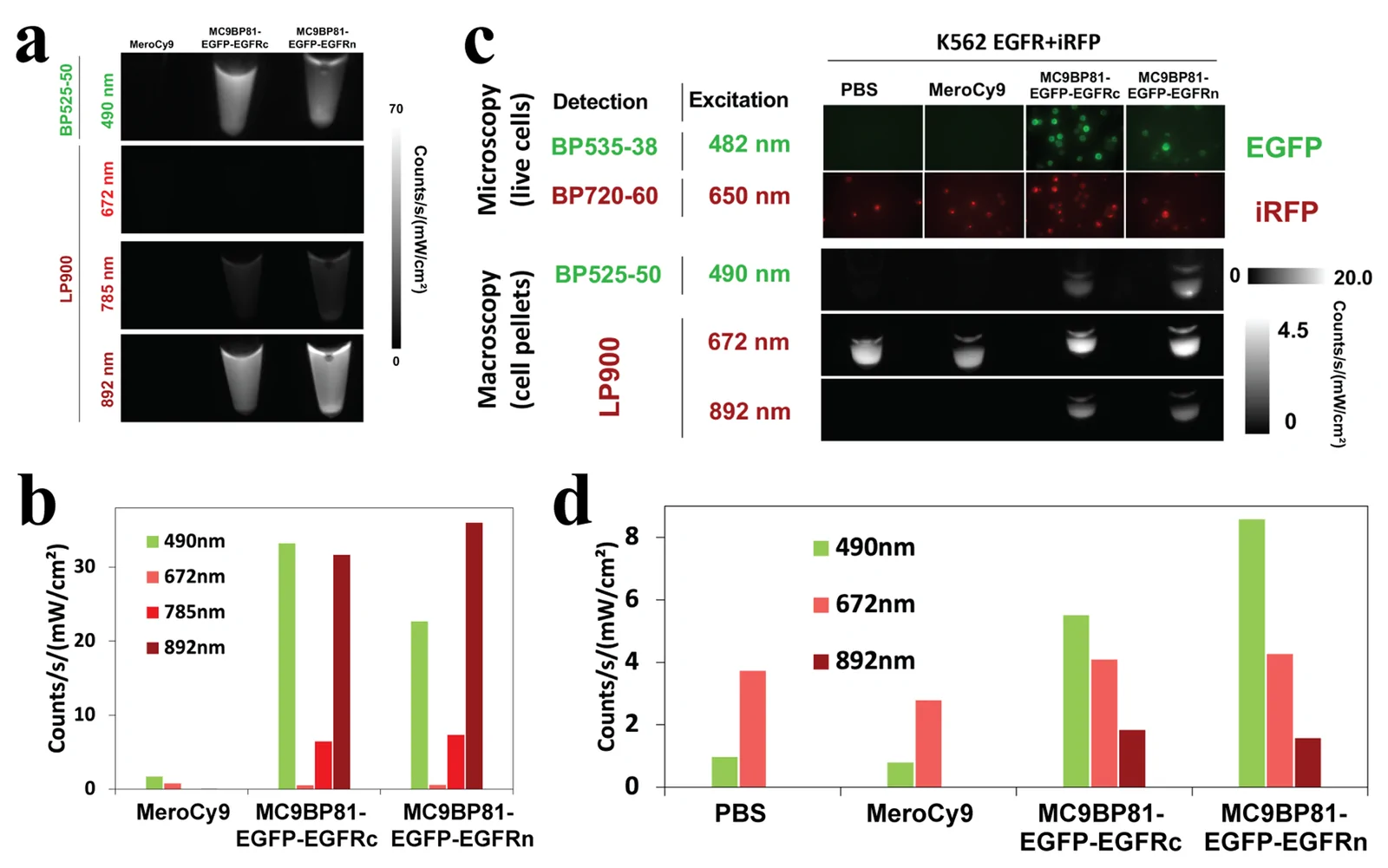
SWIR imaging using MC9BP81. (**a-b**) Purified MC9BP81-EGFP-EGFRc and MC9BP81-EGFP-EGFRn fusion proteins can be readily chromophorylated with MeroCy9 as observed using SWIR imaging. Both constructs exhibited bright fluorescence when a 900 nm long-pass filter was used. (**c-d**) After incubation with chromophorylated MC9BP81-EGFP-EGFRc and MC9BP81-EGFP-EGFRn proteins, the EGFR and iRFP720 expressing K562 cells displayed a clear fluorescence signal when measured using a 900 nm long-pass filter. Zoomed-in cell images showing membrane localization after labeling with chromophorylated MC9BP81-EGFP-EGFRc protein are shown in Figure S12e. (**b**,**d**) Measurements from different samples show good agreement (data are from a single measurement).

To explore the use of our NIR design in live-cell imaging, we first removed potential post-translational modification sites from MC9BP72 near the binding pocket entrance by substituting four serine and threonine residues with either valine or asparagine, creating MC9BP81 (see Supporting Information for protein sequences). MC9BP81 retained covalent binding to MeroCy9 and exhibited a red shift in absorption, as confirmed by ESI-MS and absorption spectra (Figures S10 and S11). We fused MC9BP81 to a designed epidermal growth factor receptor (EGFR) binding protein, EGFRc_mb (hereafter EGFRc) or EGFRn_mb (hereafter EGFRn)^43^, and EGFP. These constructs could be readily chromophorylated with MeroCy9 as observed by absorption spectroscopy (Figure S12b) and SWIR imaging (Figure 4a–b). K562 cells expressing EGFR and iRFP720^44^ as a reference were incubated with chromophorylated purified MC9BP81-EGFP-EGFRc or MC9BP81-EGFP-EGFRn proteins. The cells displayed a clear fluorescence signal when measured using a 900 nm long-pass filter whereas K562 cells not expressing EGFR showed no detected signal (Figures 4c,d and S12d,f). The membrane localization of the construct was confirmed by fluorescence imaging of the EGFP reporter (Figure S12e). Fixation of the cells did not affect the fluorescent signal (Figure S12c).

To evaluate the potential of MC9BP81 for *in vivo* imaging, we encapsulated iRFP720 expressing K562 cells labeled with chromophorylated MC9BP81-EGFP-EGFRc in alginate beads^45^ (Figure S13a) and implanted them intraperitoneally (Figure S13b) in FOXn1^nu^ mice at a cell concentration of 5000 cells/μ;L (~ 565 cells per 600 μ;m diameter bead). Imaging of both the control iRFP720 signal (672 nm excitation) and MC9BP81-EGFP-EGFRc-MeroCy9 signal (892 nm excitation) showcased the advantage of our custom designed SWIR reporter in visualizing cells deep within tissue (Figure S13b). Despite the strong expression of iRFP720, the control signal (Figure S13a) was not sufficient to reliably delineate the beads (Figure S13b). In contrast, the MC9BP81-EGFP-EGFRc-MeroCy9 signal allowed clear identification of the beads, likely due to reduced tissue autofluorescence at longer wavelengths.

## Discussion

Our designs illustrate the power of combining computational protein design with organic synthesis to generate new fluorogenic proteins with distinct spectral properties across a broad wavelength range. This approach is particularly valuable for creating proteins that operate beyond the visible light regime. Our custom-designed MeroCy7 binding protein, MC7BP34, surpasses all fluorescent proteins in the FPbase database with similar spectral profiles in terms of brightness (Figure S4b), and exhibits no cross-reactivity with the current self-labeling HaloTag and SNAP-tag proteins. The high brightness likely arises from a compact, well-designed dye-binding pocket within the protein that shields the dye from solvent and restricts its conformational flexibility. This design expands the palette for imaging applications in the far-red and near-infrared regions, and can seamlessly be integrated into most imaging setups with little or no necessary adjustments. The MeroCy9 binding proteins, MC9BP72 and MC9BP81, are the first *de novo* designed proteins that have emission profiles extending into the SWIR region, and have promising *in vivo* imaging sensitivity and depth compared with the previously described iRFP720 (Figure S13b). iRFP720, relying on the endogenous pigment biliverdin for fluorescence, is among the fluorescent proteins with the longest emission wavelengths (Figure S4). However, its peak emission is below 750 nm, and its brightness is suboptimal compared with other more blue-shifted genetically encoded fluorescent labels. Our approach provides a practical way to transcend these limitations by designing fluorescence-activating binding proteins for synthetic dyes.

The distinct spectral profiles of our two designed protein-dye pairs suggest they could be used concurrently in imaging applications. MC7BP34 does not bind to MeroCy9 even at high protein and dye concentrations. Although MC9BP81 can bind to MeroCy7, the local protein environment does not effectively activate fluorescence in the far-red region (Figure S14). The brightness of our designed NIR protein in the SWIR range is still not on par with that of ICG (Figure S12a), a typical brightness benchmark for SWIR probes^46^, but this could likely be substantially improved through directed evolution. While the protein-bound MeroCy9 dye is stable, the unbound dye gradually loses color in aqueous environments, which limits the broad applications of our designed proteins (Figures S15 and S16). To fully exploit this biological window for deep tissue imaging, further improvements in binding specificity and stability under physiological conditions will likely be required by modifying the dye molecular structure, incorporating reversible aldehyde protection, and/or further optimization of the designed protein.

Our *de novo* designed proteins provide valuable tools for deep tissue imaging and serve as prototypes for the future development of SWIR fluorescent probes. Our combined computational protein design - organic synthesis approach can be extended to address further unmet challenges. Optical imaging modeling in biological tissues suggests that fluorophores emitting around 1300 nm should provide superior performance in biomedical applications^1,3^. These fluorophores could enhance the signal-to-noise ratio by several orders of magnitude in tissues with a high hemoglobin-to-water ratio, such as blood, compared to fluorophores that fluoresce around 800 nm^3^. By designing binding proteins for dyes with an extended methine backbone or appropriate aryl fusion, it should be possible, by activating the fluorescence of the target dye, to create protein probes that are optimized to harness this biological window for deep tissue imaging. Unlike GFP, our designed proteins have an all α-helical secondary structure, which should allow for conversion into transmembrane proteins. This lays the groundwork for the development of membrane-integrated SWIR sensors to visualize electric signal processing within biological systems, particularly in neurobiological research.

## Supplementary Material

Supplementary Info Computational and experimental methods, sequences of designs, supplementary figures, supplementary table of crystallographic statistics, appendices of example design scripts and associated files.

Supplementary video Supplementary movie 1: Live cell imaging of MC7BP34-labeled mitochondria.
U2OS cells expressing a MC7BP34-GFP fusion targeted to mitochondria incubated with 0.3 μ;M MeroCy7 for 10 min then dye was chased for 15 min before imaging by spinning disk confocal microscopy (single plane). Scale bar: 10 μm.

## Data Availability

Coordinates and structure files have been deposited to the Protein Data Bank with accession codes 8U5W (WSHC5), and 8U1A (MC7BP34).
