# Supplementary material for: De novo Design of Near-Infrared Fluorescence-activating Proteins
Source: J Am Chem Soc. Author manuscript; Available in PMC 2026 Sep 10. (PMC13560888; doi:10.1021/jacs.5c19594)
Supplement: Supplementary file 1 [file NIHMS2203894-supplement-Supplementary_Info.docx]

***De novo Design of Near-Infrared Fluorescence-activating Proteins***

Yulai Liu^1^ ‡, Bernardo A. Arús^2,3^ ‡, Kanuj Mishra^4^ ‡, Taolin Wang^5,6^ ‡, Kun Zhang^5,7^, Michael P. Luciano^8^, Venu G. Bandi^8^, Akaash Kumar^9^, Ziyu Guo^10^, Yun Guan^10^, Matthew J. Bick^1^, Miaomiao Xu^1,11^, Jakob Lingg^3^, Jessica Bae^1^, Alex Kang^1^, Stacey R. Gerben^1^, Asim K. Bera^1^, Joshua C. Vaughan^10,12^, James D. Manton^9^, Emmanuel Derivery^9^, Martin J. Schnermann^8^, Andre C. Stiel^4,13, 14^, Oliver T. Bruns^2,3^, Chunfu Xu^1,5,6,15^ ‡ ^*^, David Baker^1,15 *^

1 Institute for Protein Design and Department of Biochemistry, University of Washington, Seattle, WA 98195, USA.

2 German Cancer Research Center (DKFZ), Heidelberg 69120, Germany; National Center for Tumor Diseases (NCT/UCC), Dresden 01307, Germany; Medizinische Fakultät and University Hospital Carl Gustav Carus, Technische Universität Dresden, Dresden 01062, Germany; Helmholtz-Zentrum Dresden-Rossendorf (HZDR), Dresden 01328, Germany.

3 Helmholtz Pioneer Campus, Helmholtz Zentrum München, Neuherberg 85764, Germany.

4 Institute of Biological and Medical Imaging, Bioengineering Center, Helmholtz Zentrum München, Neuherberg 85764, Germany.

5 National Institute of Biological Sciences, Beijing 102206, China.

6 Tsinghua Institute of Multidisciplinary Biomedical Research, Tsinghua University, Beijing 102206, China.

7 College of Life Sciences, Beijing Normal University, Beijing 100875, China.

8 Chemical Biology Laboratory, Center for Cancer Research, National Cancer Institute, Frederick, MD 21702, USA.

9 MRC Laboratory of Molecular Biology, Francis Crick Avenue, Cambridge CB2 0QH, UK.

10 Department of Chemistry, University of Washington, Seattle, WA 98195, USA.

11 School of Geographic and Biologic Information, Nanjing University of Posts and Telecommunications, Nanjing 210023, China.

12 Department of Neurobiology and Biophysics, University of Washington, Seattle, WA 98195, USA.

13 Institute of Biophysics and Physical Biochemistry, Regensburg Center for Biochemistry, University of Regensburg, D-93040 Regensburg, Germany.

14 Regensburg Center for Ultrafast Nanoscopy (RUN), University of Regensburg, D-93040 Regensburg, Germany.

15 Howard Hughes Medical Institute, University of Washington, Seattle, WA 98195, USA.

‡These authors contributed equally.

*Corresponding authors:

Chunfu Xu, Email: xuchunfu@nibs.ac.cn;

David Baker, Email: dabaker@uw.edu

**Contents**

Computational methods	3

Experimental materials and methods	5

Supplementary figures	24

Crystallography data	42

Appendices	43

References	57

### **Computational methods**

Design of helical bundle oligomers

The helical bundle oligomeric scaffolds were designed by combining parametric backbone generation and RosettaDesign sequence calculations as described previously[^1,2^](https://paperpile.com/c/GVLBeS/QKff+bWr2).

Design of MeroCy7 and MeroCy9 binding proteins.

The oligomeric scaffolds were linked using short loops to form single-chain proteins via the Rosetta ConnectChainMover[^3^](https://paperpile.com/c/GVLBeS/mhhP). Given that dye molecules covalently bond to activated lysine residues, we treated the dye-lysine conjugates as noncanonical amino acids[^4^](https://paperpile.com/c/GVLBeS/eq4p) (NCAAs) and allowed Rosetta to place these NCAAs at different selected pocket positions—though only one NCAA was permitted per design calculation. This approach bypassed the usual global docking phase required to place the small molecule in the preferred binding sites of the protein scaffolds. As a result, we could leverage Rosetta’s sequence design machinery without the need for additional optimization.

Design protocols.

See appendices.

Sequences of designed proteins

| WSHC5 | TRRKQEMKRLKKEMEKIREETEEVKKEIEESKKRPQSESAKNLILIMQLLINQIRLLALQIRMLALQLQE |
| --- | --- |
| MC7BP34 | SEREEELKRLKKEQEKIREEIEEVKKEIEESKKRPQSESQKNFILSLQLFISMLRLKLLWSRALALQLQRERLTDTDEVDRRREQELKRLKKELEKLREETEEVKKEIEESKKRPQSESLKNIILINQLLILVIRSEYLIIRNLISQLQAQTNPNNTRLKQEQKRSKKEQEKIREELEEVKKEIEESKKRPQSESAKNFILMAQSLISLIRLLALITRALNLQLQARRDGPNEELEQELKRLKKEVEKIREEQEEVNKEIEESKKRPQSESLKNFILLAQLISSMVRLWELIIRILQLQLQKRKGINEDELREELKRLKKETEKIREETEEVKKEIEESKKKASQTKEIILMLQLEIAWIRSLLSIIRLLKLQLEQIDK |
| MC9BP72 | TEDELRKLRKALEEAEKKLYKLEDSLRRLEEMAKTDDDPKAQSLAVIAESKTLIAESLLIWAKSVLLSSRANRGHDNKHEAEYELRKLRRELERAEEKAKRLEEKARYLEEIAKTDDDPKAQSLVQIALSLVLLAESLFVIATSFVISSYLNIQPPTEEAEKELRKLRERLKKAEEKLKELEKSAYKLEEIAKTDDDPKAQSLFQIAQSWTAFAISLWIIAVSLLISSELNLKGPDDKLEEELRKLREQLKELEKKLRELEKSARKLEEILKTDDDPKAQSLAQIALSNTLLAISFTIEATSLLISSWLNLKGSDKKLEEELRKLRRELEEAEKLLKEAEESVKYLEEMAKTDDDPKVQSLFLAAESNTIIALSIFIIATSLTLSSYARLTGPTEEAEREIRYLRELLKEAEKKLKELEKKAKRLEEMLKTDDDPKAQSLAQFAESEVLIAESIWVAAVSFLLSTRNT |
| MC9BP81 | TEDELRKLRKALEEAEKKLYKLEDSLRRLEEMAKTDDDPKAQSLAVIAESKTLIAESLLIWAKSVLLVSRANRGHDNKHEAEYELRKLRRELERAEEKAKRLEEKARYLEEIAKTDDDPKAQSLVQIALSLVLLAESLFVIATSFVISSYLNIQPPTEEAEKELRKLRERLKKAEEKLKELEKSAYKLEEIAKTDDDPKAQSLFQIAQSWTAFAISLWIIAVSLLISSELNLKGPDDKLEEELRKLREQLKELEKKLRELEKSARKLEEILKTDDDPKAQSLAQIALSNTLLAISFTIEATSLLISSWLNLKGSDKKLEEELRKLRRELEEAEKLLKEAEESVKYLEEMAKTDDDPKVQSLFLAAESNTIIALSIFIIATSLTLVSYARLTGPTEEAEREIRYLRELLKEAEKKLKELEKKAKRLEEMLKTDDDPKAQSLAQFAESEVLIAESIWVAAVSFLLVTRNN |

### **Experimental materials and methods**

Reagents

Chemicals used were of the highest grade commercially available and were purchased from Sigma-Aldrich (St. Louis, MO, USA), Invitrogen (Carlsbad, CA, USA), or Qiagen (Hilden, Germany). Detergents were from Anatrace (Maumee, OH, USA).

Synthesis of MeroCy7

**MeroCy7:** 1,2,3,3-Tetramethyl-3*H*-indolium iodide (50 mg, 0.16 mmol), N-((1Z,2E,4E,6E)-7-(phenylamino)hepta-2,4,6-trien-1-ylidene)benzenaminium perchlorate[^5^](https://paperpile.com/c/GVLBeS/dDP9) (68 mg, 0.18 mmol, 1.1 eq) and NaOAc (26 mg, 0.32 mmol, 2 eq) were suspended in acetic anhydride (2 mL) in a 1-dram vial equipped with a magnetic stir bar and stirred at ambient temperature for 30 min, during which time the suspension became homogeneous and the color changed to magenta. The reaction mixture was precipitated into diethyl ether (40 mL) in a 50 mL conical tube and vortexed until a purple precipitate formed. The reaction mixture was centrifuged at 5000 rpm for 5 min, and the supernatant discarded. The pellet was resuspended in diethyl ether (40 mL) and the process was repeated two more times. The purple pellet was dried under vacuum for 1 h then used in the next step without further purification. The crude intermediate was dissolved in MeCN (2 mL) and treated with *n*-butylamine (82 µL, 0.8 mmol, 5 eq), and the color instantly changed to yellow. LCMS analysis indicated conversion to the *n*-butyl-imine. The solvents were evaporated, and the crude material was purified by automated flash chromatography (silica-0-10% MeOH/DCM gradient) to afford the *n*-butyl-imine intermediate as a blue film (56.9 mg). The imine intermediate was dissolved in 50/50 MeCN/water (4 mL) and heated to 70 °C for 2 h and LCMS analysis indicated formation of **MeroCy7** aldehyde. The reaction mixture was diluted with brine (10 mL) and extracted into diethyl ether (3 x 10 mL), the combined organic extract was dried over sodium sulfate and concentrated to afford **MeroCy7** (10.3 mg, 22% overall yield) as a red solid. ^1^H NMR (400 MHz, CDCl_3_) δ 9.51 (d, J = 8.1 Hz, 1H), 7.21 – 7.12 (m, 3H), 7.08 (dd, J = 14.1, 12.2 Hz, 1H), 6.89 (td, J = 7.4, 1.0 Hz, 1H), 6.82 (dd, J = 14.4, 11.4 Hz, 1H), 6.66 (d, J = 7.8 Hz, 1H), 6.31 (dd, J = 14.5, 11.4 Hz, 1H), 6.18 – 6.03 (m, 2H), 5.41 (d, J = 12.2 Hz, 1H), 3.16 (s, 3H), 1.59 (s, 6H) ppm; ^13^C NMR (100 MHz, CDCl_3_) δ 193.57, 160.31, 153.70, 145.43, 144.86, 139.12, 136.92, 128.15, 128.03, 124.84, 123.90, 121.77, 120.46, 106.60, 96.88, 77.40, 46.16, 29.35, 28.49 ppm; HRMS (ESI – H^+^) 280.1696 calculated for C_19_H_21_NO, found 280.1685.

Synthesis of MeroCy9

**S1:** To a microwave reaction vial equipped with a magnetic stir bar was added 1,2,3,3-Tetramethyl-3*H*-indolium iodide (952 mg, 3.16 mmol, 1 eq), 1,8-dichloro-7-(hydroxymethylene)-3,4,4a,5,6,7-hexahydronaphthalene-2-carbaldehyde (1.3 g, 5.05 mmol, 1.6 eq)[^6^](https://paperpile.com/c/GVLBeS/JDhFT) and sodium acetate (259 mg, 3.16 mmol, 1 eq). After flushing with argon, ethanol (7 mL) was added. The reaction mixture was stirred at room temperature for 2.5 h. Evaporated the solvent, and the reaction mixture was diluted with dichloromethane. Washed the reaction mixture with water and dried through sodium sulfate and filtered. Evaporated the solvent and purified by normal-phase chromatography (40 g silica column, 0→100% hexane / dichloromethane). The product-containing fractions were evaporated to afford **S1** (325 mg, 25% yield) as a red colored solid. ^1^H NMR (400 MHz, Chloroform-*d*) δ 10.36 (s, 1H), 7.65 - 7.62 (d, 1H), 7.21 - 7.17 (m, 2H), 6.92 -6.88 (t, 1H), 6.68 - 6.66 (d, 1H), 5.46 - 5.43 (d, 1H), 3.20 (s, 3H), 2.58 - 2.53 (m, 2H), 2.51 - 2.49 (d, 1H), 2.43 - 2.38 (m, 3H), 2.22 - 2.17 (m, 1H), 1.65 - 1.63 (d, 6H), 1.38 - 1.36 (m, 2H); ^13^C NMR (500 MHz, chloroform-*d*) δ 192.27, 161.01, 144.73, 144.64, 139.15, 137.30, 134.97, 128.61, 128.41, 127.80, 126.16, 121.72, 120.34, 106.35, 92.95, 46.15, 40.16, 29.73, 29.60, 29.28, 28.46, 28.06, 24.89, 24.58; HRMS (ESI – ·+) calculated for C_24_H_25_Cl_2_NO 413.1308, observed 414.1301.

**MeroCy9:** To a microwave vial equipped with a magnetic stir bar was added Compound **S1** (95 mg, 0.22 mmol, 1 eq), 1,2-bis(4,4,5,5-tetramethyl-1,3,2-dioxaborolan-2-yl)benzene (227.17 mg, 0.68 mmol, 3 eq), Pd(PPh_3_)_4_ (26.52 mg, 0.02 mmol, 0.1 eq) and K_3_PO_4_ (97.41 mg, 0.45 mmol, 2 eq). The vessel was sealed and flushed with argon. 1 mL of 5:1 DMF: H_2_O was added, and the reaction was heated to 100 °C for 15 h. LC/MS analysis indicated the formation of a new peak (m/z^+^ 582), which is the product of single coupling. The reaction was cooled and diluted with dichloromethane and filtered through celite. The dichloromethane layer was washed with water and dried through Na_2_SO_4_ and filtered. The solvent was transferred to the microwave reaction vial and evaporated. To the vial was added Pd(PPh_3_)_4_ (26.52 mg) and potassium phosphate tribasic (97.41 mg). The vessel was sealed and flushed with argon. 1 mL of 3:2 ratio of DMF: 250mM pH 9.0 PBS was added, and the reaction was heated to 100 °C overnight (~ 15 h). LC/MS analysis then indicates a new major product **MeroCy9** (m/z^+^ 420). The reaction was cooled and diluted with dichloromethane and filtered through celite. The dichloromethane layer was washed with water and dried through sodium sulfate and filtered. Purified by normal-phase chromatography (12 g SiO_2_ column, 0→100% hexane / dichloromethane). The product-containing fractions were evaporated to afford **MeroCy9** (47.59 mg, 49% yield) as a blue solid. ^1^H NMR (400 MHz, Chloroform-*d*) δ 10.81 (s, 1H), 7.84 - 7.82 (d, 1H), 7.74 - 7.73 (d, 1H), 7.67 – 7.63 (m, 2H), 7.38 – 7.34 (m, 2H), 7.20 - 7.17 (m, 2H), 6.91 – 6.87 (t, 1H), 6.66 - 6.66 (d, 1H), 3.22 (s, 3H), 2.60 - 2.53 (m, 2H), 2.46 - 2.41 (m, 1H), 2.34 - 2.33 (m, 1H), 2.13 - 2.12 (m, 3H), 1.73 – 1.71 (d, 6H), 1.30 – 1.27 (m, 2H); ^13^C NMR (500 MHz, chloroform-*d*) δ 188.98, 158.95, 148.67, 144.94, 142.91, 140.66, 140.21, 138.98, 137.92, 136.08, 135.09, 128.66, 127.85, 125.73, 125.71, 125.37, 124.39, 121.65, 121.19, 120.02, 106.21, 93.63, 45.77, 33.92, 30.34, 29.98, 29.28, 28.21, 28.18, 26.32, 23.54; HRMS (ESI – H^+^) calculated for C_30_H_29_NO 420.2322, observed 420.2296.

NMR spectra

Cloning and expression of designed proteins

To obtain the genes encoding the designed proteins, the amino acid sequences of the designs were reverse translated to DNA sequences using DNAworks[^7^](https://paperpile.com/c/GVLBeS/KGwx) (https://github.com/davidhoover/DNAWorks). The codons were optimized for *E. coli* expression. Synthetic genes were obtained from Genscript Inc. (Piscataway, N.J., USA) or IDT (Coralville, Iowa, USA) and cloned into the pET29b expression vector via NdeI/XhoI restriction sites. The plasmids were transformed into chemically competent *E. coli* Lemo21(DE3) cells (NEB, Ipswich, MA). Gene expression was facilitated by growing pre-cultures in Terrific Broth (TB) medium with a final concentration of 50 µg/mL kanamycin overnight at 37 °C. 10 mL pre-cultures were used to inoculate 1 L of autoinduction medium[^8^](https://paperpile.com/c/GVLBeS/ITuI), again containing 50 µg/mL kanamycin for plasmid selection. The cultures were grown at 37°C overnight for protein expression. Cells were harvested by centrifugation.

Purification of designed proteins

Cells were resuspended and homogenized in lysis buffer containing 50 mM Tris-HCl pH 8.0 and 300 mM NaCl 20 mM imidazole, and lysed by sonication in the presence of DNase and EDTA-free protease inhibitor tablet (ThermoFisher Scientific). Lysates were cleared by centrifugation at 4°C 18,000 g for at least 30 min and applied to Ni-NTA (Qiagen) columns pre-equilibrated in the lysis buffer. The column was washed with 5-10 column volumes (CV) of wash buffer (50 mM Tris, 300 mM NaCl, 30 mM imidazole, pH 8.0), followed by 5 CV of high-salt wash buffer (50 mM Tris, 1 M NaCl, 30 mM imidazole, pH 8.0), and then 5 CV of high-imidazole wash buffer (50 mM Tris, 300 mM NaCl, 60 mM imidazole, pH 8.0). Protein was eluted with 50 mM Tris, 300 mM NaCl, 500 mM Imidazole, pH 8.0. After concentration, proteins were further purified by SEC (Superdex 200 increase 10/300 GL or HiLoad 16/600 Superdex 75 pg; Cytiva Life Sciences) using 1x phosphate-buffered saline (PBS), pH 7.4.

*In vitro* dye binding

MeroCy7 binding procedure: we prepared a MeroCy7 stock solution by dissolving the dye in methanol, achieving a final concentration of 1.0 mM. This stock solution was stored at -20 °C. For each binding test, we freshly made either a 50x or 100x dye solution in 0.15% n-Dodecyl-β-D-Maltopyranoside (DDM, from Anatrace Products, LLC.) or 0.2% n-Decyl-β-D-Maltopyranoside (DM, from Anatrace Products, LLC.). These detergents were used to stabilize the dye in an aqueous setting. We then mixed this dye solution with purified protein solutions, either in equimolar amounts or with a slight excess of one component, depending on the requirements of subsequent applications. After mixing, the dye-protein samples were left to sit at room temperature for 30 min to ensure complete binding.

MeroCy9 binding procedure: we prepared a MeroCy9 stock solution by dissolving the dye in DMSO, achieving a final concentration of 1.5 mM. This stock solution was stored at -80 °C. For each binding test, we freshly made a 50x dye solution in 0.15% n-Dodecyl-β-D-Maltopyranoside (DDM, from Anatrace Products, LLC.). We then mixed this dye solution with purified protein solutions, either in equimolar amounts or with a slight excess of one component, depending on the requirements of subsequent applications. After mixing, the dye-protein samples were left to sit at room temperature for at least 4 h (or overnight) to ensure complete binding. When needed, unbound dye was removed by passing the chromophorylated samples through SEC columns.

Directed evolution of MeroCy7 binding proteins

Given the protein's large size, construction of site-saturation mutagenesis library was limited to residues surrounding the dye molecule in the design model. The library was constructed using an efficient one-step PCR protocol[^9^](https://paperpile.com/c/GVLBeS/LSQR). We then transformed the PCR product into XL-1 Blue competent cells (Agilent Technologies, Inc.) and inoculated them in Terrific Broth (TB). After an overnight incubation at 37 °C, we extracted the plasmids using a Qiagen miniprep kit. These purified plasmids were subsequently transformed into Lemo21(DE3) competent cells (New England Biolabs) and inoculated on a Luria–Bertani (LB) plate. Throughout these steps, we maintained separation between the sublibraries corresponding to each residue position. From every sublibrary, 96 colonies were picked and cultivated in 1 mL of autoinduction medium[^8^](https://paperpile.com/c/GVLBeS/ITuI) in a deep 96-well plate. After overnight incubation at 37°C, cells were then pelleted using centrifugation and resuspended in lysis buffer containing PBS with 2 mg/mL lysozyme from chicken egg white (Sigma). The plate was vigorously shaken for 12-24 h at 37 °C. The lysate was then cleared by centrifugation. 100 μL of each clarified lysate was then transferred to a 96-well assay plate and MeroCy7 was added to a final concentration of 200 nM to each well. After sitting at room temperature for 30 min, fluorescence spectra were measured on a Synergy Neo2 hybrid multi-mode reader (BioTek). Mutants which showed improved fluorescence emission intensity were then selected and sequenced. Combinatorial libraries were then generated by combining the beneficial mutations using the same PCR protocol and screened as described above.

Absorbance measurement and extinction coefficient determination of MC7BP34

MC7BP34 protein solutions with concentrations of 0.625, 1.25, 2.5, and 5 μM were prepared in an aqueous buffer containing 20 mM Tris-HCl pH 8 and 150 mM NaCl. Absorbance measurements were recorded at the absorption peak wavelength, 663 nm, with a Denovix UV-Vis spectrophotometer, in a quartz cuvette with 1 cm path length. The molar extinction coefficient was calculated from the slope of the linear fit of absorbance vs. concentration.

Absolute quantum yield measurement of MC7BP34

The absolute quantum yield was measured using a Hamamatsu C9920-02, A10094 Quantum Efficiency Measurement System at the Photonic Research Center in the UW Department of Chemistry. The instrument is equipped with an integrating sphere, an ultra-high sensitivity CCD sensor, and a Xenon light source. It was operated with a 6 nm excitation bandwidth and a 1 nm interval.

The MC7BP34 protein solution was diluted to 0.625 μM in an aqueous buffer containing 20 mM Tris-HCl pH 8 and 150 mM NaCl. The MC7BP34 protein solution and a reference blank buffer were measured at the same conditions in a 1 mL quartz cuvette with 1 cm path length and using an excitation wavelength centered at 620 nm. The absolute quantum yield Φ was calculated according to Φ = *f*_em_/*f*_abs_ where *f*_em_ is the emitted photon flux and *f*_abs_ is the absorbed photon flux. The same procedure was used to measure the absolute quantum yield values of two spectrally-similar control samples (Alexa Fluor 680, Cy5) which agreed well with literature values[^10,11^](https://paperpile.com/c/GVLBeS/hlsu+fypV).

Circular dichroism (CD) measurement

CD wavelength-scan measurements were made on J-1500 Circular Dichroism Spectrophotometer (Jasco). Protein solutions were prepared in an aqueous buffer containing 20 mM Tris-HCl (pH 8) and 150 mM NaCl at 0.4 mg/mL. Wavelength-scan spectra were recorded from 200 nm to 260 nm (1 nm increments). Temperature melts were conducted in 10 °C steps from 25 °C to 95 °C (heating rate 1 °C/min). The full spectra were measured again after the samples were cooled down to 25 °C.

X-ray crystallography

Protein samples were prepared in a buffer containing 20 mM Tris-HCl (pH 8) and 150 mM NaCl. For WSHC5, the protein was concentrated after elution from the SEC column and subjected to crystallization trials. For MC7BP34, the proteins were mixed with the dyes in a 1:1.5 molar ratio. The protein-dye complexes were incubated at 4 °C overnight and loaded onto the SEC column to remove the free dye molecules. The fractions containing the complex were pooled and concentrated to 10-20 mg/mL for crystallization trials. Crystallization experiments for the WSHC5 and MC7BP34 proteins were conducted using the sitting drop vapor diffusion method. Crystallization trials were set up in 200 nL drops using the 96-well plate format at 20°C. Crystallization plates were set up using a Mosquito LCP from SPT Labtech, then imaged using UVEX microscopes from JAN Scientific. Diffraction quality crystals formed in 0.2 M Ammonium acetate, 0.1 M BIS-TRIS pH 5.5, 25% w/v Polyethylene glycol 3,350 for WSHC5 and in 0.2 M Trimethylamine N-oxide dihydrate, 0.1 M Tris pH 8.5, 20% w/v Polyethylene glycol monomethyl ether 2,000 for MC7BP34.

Diffraction data were collected at ALS-ENABLE beamlines for WSHC5 and at the Advanced Photon Source beamline 24ID-C for MC7BP34. X-ray intensities and data reduction were evaluated and integrated using XDS[^12^](https://paperpile.com/c/GVLBeS/mG2u) and merged/scaled using Pointless/Aimless in the CCP4 program suite[^13^](https://paperpile.com/c/GVLBeS/LWbs). Structure determination and refinement starting phases were obtained by molecular replacement using Phaser[^14^](https://paperpile.com/c/GVLBeS/hQgD) with the designed model structure. Following molecular replacement, the models were improved using phenix.autobuild^[15](https://paperpile.com/c/GVLBeS/dWjo)^. Structures were refined in Phenix[^15^](https://paperpile.com/c/GVLBeS/dWjo). Model building was performed using COOT[^16^](https://paperpile.com/c/GVLBeS/eRhN). The final model was evaluated using MolProbity[^17^](https://paperpile.com/c/GVLBeS/tFwB). Data collection and refinement statistics are recorded in Table S1. Data deposition, atomic coordinates, and structure factors reported for the protein in this paper have been deposited in the Protein Data Bank (PDB), http://www.rcsb.org/ with accession codes 8U5W and 8U1A.

Live cell imaging of MC7BP34

For expression of MC7BP34 expression in mammalian cells, a gene fragment encoding MC7BP34 flanked in N-terminus by a Mito-Tag—the first 34 residues of the Mas70p protein, shown to efficiently relocalize proteins to mitochondria in mammalian cells[^18^](https://paperpile.com/c/GVLBeS/WC2j)— and a C-terminal EGFP was synthesized by Twist Bioscience and cloned into a modified pTwist vector plasmid allowing mammalian expression under an EF1a promoter.

U2OS FlipIn Trex cells (a gift from S. C. Blacklow) were cultured in DMEM (Corning) supplemented with 10% fetal bovine serum (FBS, Gibco) and 1% penicillin–streptomycin (Gibco) at 37 °C with 5% CO_2_. Cells were transfected with Lipofectamine 3000 (Invitrogen) according to the manufacturer’s instructions, and imaged after one day of expression. Cell lines were not authenticated. Cells were screened for mycoplasma using MycoAlert Mycoplasma Detection Kit (Lonza).

On the day of the experiment, transfected cells were plated on glass-bottom dishes (World Precision Instruments, FD35) coated with fibronectin (Sigma, F1141, 50 μg/mL in PBS), for 2 h at 37 °C in DMEM-10% serum. Cells were then pulsed with MeroCy7 dye by incubating them with pre-warmed, filtered L15 medium (Gibco), enriched with 4.5 g/L glucose, 10% FBS and 20 mM HEPES (Gibco), and 0.3 µM MeroCy7 and 0.003% DDM (from an intermediary MeroCy7 stock solution of 15 µM MeroCy7 and 0.15% DDM in PBS). After 15 min at 37 °C, unbound dye was washed extensively and imaging was performed in L15 medium, enriched with 4.5 g/L glucose, 10% FBS and 20 mM HEPES.

We noticed that the MeroCy7 dye tended to accumulate in intracellular compartments, in particular lysosome- and mitochondria-like structures in untransfected cells (see Figure S3). For this reason, the MeroCy7 dye cannot be kept in the imaging medium but should rather be washed in a pulse-chase experimental design. When using such pulse-chase experiments, the dye non-specifically accumulating in compartments quickly washes away and specific MC7BP34 is observed (see Figure 2g). Non-specific uptake of the dye has been observed in several cell lines, and thus the exact pulse-chase conditions might need some adaptation for some cell lines.

Imaging was performed using a custom spinning disk confocal instrument composed of a Nikon Ti stand equipped with a perfect focus system, a fast Z piezo stage (ASI) and a PLAN Apo Lambda 1.4 NA 60× objective, and a spinning disk head (Yokogawa CSUX1). Images were recorded with a Photometrics Prime 95B back-illuminated sCMOS camera run in pseudo global shutter mode and synchronized with the spinning disk wheel. Excitation was provided by 488 and 630 nm lasers (Coherent OBIS mounted in a Cairn laser launch) and imaged using dedicated single-bandpass filters for each channel mounted on a Cairn Optospin wheel (Chroma 525/50 for GFP and Chroma 647lp for MC7BP34). The temperature was kept at 37 °C using a temperature control chamber (MicroscopeHeaters.Com). The system was operated by MetaMorph.

Multicolor microscopy imaging in live HEK293T cells

For three-color imaging experiments, pcDNA3.1(+) was used as the expression vector for transient co-expression of HaloTag7, SNAP-tag (the fast variant E30R, SNAPf) and MC7BP34. HaloTag7 was fused to the C-terminus of the outer mitochondrial membrane protein Tomm20 for mitochondrial tagging (Tomm20-HaloTag7), SNAPf was fused to the N-terminus of the tubulin protein for microtubule tagging (SNAPf-tubulin), and MC7BP34 was fused to the C-terminus of the histone H2B for cell nucleus tagging (H2B-MC7BP34).

HEK293T cells were cultured in DMEM (Gibco) supplemented with 10% HyClone FetalClone II (FC-II, Cytiva SH30066.03) and 1% penicillin-streptomycin (Gibco) at 37 °C with 5% CO_2_. Cells were preseeded into CELLview glass-bottom cell culture slides (Greiner Bio-One #543079) at a density of 50,000 cells per well. The following day, cells were transfected using Lipofectamine 3000 according to the manufacturer’s instructions with all three plasmids mixed at equimolar ratios.

Chromophore labeling was performed on the next day after transfection. SNAP-Cell Oregon Green substrate (NEB S9104S) was dissolved in DMSO at a concentration of 1 mM to make the stock solution. The SNAP substrate was diluted from the stock solution in the labeling solution (Gibco FluoroBrite DMEM supplemented with 10% FC-II) to a final concentration of 3 µM. The cell culture medium was substituted with this labeling solution containing the SNAP substrate and incubated at 37 °C/5% CO_2_ for 30 min. Following the incubation, the cells were washed twice with the labeling solution and incubated in the labeling solution for 1 h to allow the unreacted substrates to diffuse out of the cells. Subsequently, one vial (1 nmol) of the Janelia Fluor 549 HaloTag Ligand (JF549, Promega HT1020) was resuspended with 1 mL of the labeling solution (1 µM ligand concentration) and further diluted to a final concentration of 200 nM for labeling. The cells were exposed to this JF549-containing solution (100 µL per well) and incubated at 37 °C/5% CO_2_ for 15 min. The MeroCy7 dye was diluted to the labeling solution supplemented with 0.015% DDM at a concentration of 5 µM to prepare the 5x working solution. A volume of 25 µL of this solution was added to the cells, achieving a final MeroCy7 concentration of 1 μM with 0.003% DDM. After incubation for 15 min at 37 °C/5% CO_2_, cells were washed using the labeling solution three times, with a 1-minute interval between each wash, and finally immersed in HEPES-buffered solution (10 mM HEPES, 140 mM NaCl, 5 mM KCl, 2 mM MgCl_2_, 2 mM CaCl_2_, 10 mM glucose, 15 mM sucrose, pH 7.4 adjusted by NaOH) for imaging on the DeltaVision OMX SR imaging system (GE Healthcare, v4.5.10296-1) equipped with an Olympus Apo N TIRF 60x/1.49 Oil objective. The excitation and emission detection wavelengths were 488 nm/528 nm, 568 nm/609 nm, and 640 nm/683 nm for the green, orange, and far-red channels, respectively. Sequential ring-TIRF operating mode was used for better visualization of microtubules and mitochondria. The incident angles of the excitation beams were determined and corrected automatically by the OMX imaging system, while the beam path of the far-red channel was not adjusted (given that the volume of cell nuclei spans across all the acquired stacks along the Z distance). Z-stacks were recorded with 0.25 μm steps. For image visualization following image acquisition, Z-stack projections by maximum intensity of the captured images were generated for all fluorescent channels in Fiji. The Z-stacked images were subsequently adjusted for contrast and gamma using Napari image viewer and plotted using Matplotlib.

K562 cell labeling and alginate beads preparation

K562 cells, serving as the control group, as well as K562 cells expressing EGFR on their cell surface and iRFP720, were cultured following the recommended procedures for K562 cell culture provided by ATCC. In brief: cells were maintained in Iscove's Modified Dulbecco's Medium (IMDM media, Sigma-Aldrich, I6529). The medium was supplemented with 10% fetal bovine serum obtained (Invitrogen) and penicillin (100 U/mL) and streptomycin (100 μg/mL). Cells were cultured under controlled conditions at 37 °C with a 5% CO_2_ atmosphere. For labeling, 2 x 10^6^ cells were treated with purified, chromophorylated MC9BP81_EGFP_EGFRc or MC9BP81_EGFP_EGFRn for 1 h at 4 °C with overhead rotation. Following incubation, cells were washed three times and resuspended in 200 µL of phosphate-buffered saline (PBS).

Prior to encapsulation of iRFP720-expressing K562 cells within alginate beads, we verified both the intracellular expression of iRFP and the surface binding of the MC9BP81_EGFP_EGFRc or MC9BP81_EGFP_EGFRn probe. Fluorescence microscopy confirmed signal across the expected channels: iRFP expression in K562 was detected using 650 nm excitation with a BP720-60 emission filter, while the binding of the EGFP-tagged probes was validated using 482 nm excitation and a BP535-38 emission filter. Alginate beads were prepared as described before[^19^](https://paperpile.com/c/GVLBeS/zlcz). Briefly, a 4% (w/v) solution of sodium alginate was 1:1 mixed with the cells (see above). The alginate cell mixture was filled in a syringe with a 25G needle and centrifuged at 300 rpm. Beads formed by the mixture dropping into a 200 mM CaCl_2_ solution. Beads were allowed to solidify before CaCl_2_ was replaced by PBS buffer.

Macroscopic NIR-SWIR fluorescence imaging

Custom-built NIR-SWIR setups were used for the imaging experiments. These setups generally consisted of a sCMOS (PCO Edge 4.2 or Photometrics PrimeBSI) or InGaAs detector (PCO Pixelfly 1.3 SWIR), SWIR-transmissive lenses and optical filters (band- or long-pass from Thorlabs and Edmund Optics) in the detection side, and laser or LED units on the excitation side. The laser units had their outputs coupled in a 4x1 fan-out fiber-optic bundle (Thorlabs BF46LS01) of 600-μm core diameter for each optical path. The common end of the fiber bundle was introduced into an excitation cube (Thorlabs KCB1EC/M), reflected off a mirror (Thorlabs BBE1-E03), and passed through a positive achromat (Thorlabs AC254-050-B), short-pass filters for laser cleanup, and an engineered diffuser (Thorlabs ED1-S20-MD) for uniform illumination on the field of view. To excite GFP, 2x 490 nm LEDs (Thorlabs M490L4) collimated with aspheric condenser lenses (Thorlabs ACL25416U-A) and cleaned-up with 500-nm short-pass filters (Thorlabs FESH0500) were employed, with a maximum laser power density of 17 mW/cm². For iRFP720, ICG, and MeroCy9 excitation, 672, 785, and 892 nm lasers were respectively utilized (Lumics, LU0680D010-E10FH, LU0785D250-U70AN, LU0890D400-U10AF), with maximum laser power density ranging from 69 to 141 mW/cm², depending on the experiment. Laser power was measured with a Thorlabs power meter (PM100D, with sensor S130C). In addition to the samples imaged in the experiments, dark and flatfield takes were acquired. For imaging acquisition with the sCMOS detectors, microManager (MM Studio v. 2.0.0, MMCore v. 10.1.1) was used, while PCO Camware (v. 04.12/1705) was employed to acquire images with the InGaAs detector.

Macroscopic fluorescence image processing and analysis

Images were processed using Python3 and the Fiji distribution of ImageJ. Dark and flatfield frames were generated from the average of 10 frames. All takes had their corresponding dark frames (of matching exposure time) subtracted. Power density maps were generated by multiplying the flatfield frame to the maximum power density. All images from in vitro macroscopic experiments were flatfield-corrected, corrected for exposure time, and divided by their corresponding power density maps to generate counts/s/(mW/cm²). Given the three-dimensional nature of in vivo wide-field imaging, images from in vivo experiments did not undergo flatfield correction, and were therefore represented in counts/s. Image quantification was performed by drawing regions of interest (ROI) comprising the signal of each sample, and the mean value in each ROI was measured. The line profile on the in vivo beads experiment was obtained by plotting the intensity of each pixel along the line ROI.

*In vivo* macroscopic fluorescence imaging

Animal procedures were performed in conformity with the institutional guidelines as well as national regulations, and after approval by the government of Upper Bavaria. A 7-week old, female athymic nude mouse (housed at 23 °C, 45–65% humidity, with a 12/12 h light/dark cycle) was anesthetized with a ketamine/xylazine solution, and had alginate beads (prepared as described above) injected subcutaneously in the interscapular space, as well as in the intraperitoneal space with a 25G needle. Macroscopic fluorescence imaging was conducted before and after the injections with the Photometrics Prime BSI detector (binned 2x), 50-mm lens (Navitar SWIR-50), using a 950-50 nm band-pass filter (Edmund Optics), and exposure time of 2500 ms. For the interscapular images, a 892-nm laser (Lumics) was utilized as excitation source. For the intraperitoneal take, a sequence of images was acquired, and the lasers 672, 785 and 892 nm (Lumics) were individually switched on in specific frames to evaluate the emitted signal upon each laser excitation. The same 900 nm short-pass filter (Thorlabs FESH0900) was used to clean up off-peak excitation light.

Absorption spectroscopy of MeroCy9 binding proteins

Solutions of chromophorylated MC9BP81-MeroCy9 (in PBS) and ICG (in fetal bovine serum, FBS) were placed in a quartz cuvette (Hellma 115B-10-40, 10 mm path length), and had their absorption spectra acquired with a UV–VIS–NIR spectrophotometer (PerkinElmer Lambda1050+). The spectra of PBS and FBS were acquired as blank measurements, and subtracted from their corresponding fluorophore solution.

Fluorescence spectroscopy of MeroCy9 binding proteins

Fluorescence emission spectra were acquired using a liquid nitrogen-cooled InGaAs line detector (Princeton Instruments Pylon-IR 1024-1.7) connected to a Czerny-Turner spectrograph (Princeton Instruments HRS-300). A super-continuum white light laser (NKT Photonics SuperK-Extreme) filtered with a tunable filter (NKT Photonics SR-Extended-8HP LLTF Contrast) was used as excitation source. 785 nm, further filtered by a 785-10 band-pass filter (Edmund Optics), was used as the wavelength to excite both ICG and MC9BP81-MeroCy9. The photoemission was collected using a pair of silver-coated off-axis parabolic mirrors (Thorlabs MPD269-P01 and MPD269-P01-H), which focus the light onto a 400-µm multimode fiber (Thorlabs, M28L01) that was connected to the spectrograph. Two 800-nm long-pass filters (Thorlabs FELH0800) were positioned in front of the fiber to further block the excitation light source. Intensity correction takes were acquired with a calibration lamp (Thorlabs SLS201L/M). Background takes were also acquired. Background and intensity corrections were performed using Python3.

### **Supplementary figures**

**Figure S1.** Biophysical characterizations of designed fluorescent proteins. (**a**-**b**) Circular dichroism spectra for MC7BP34 (**a**) and MC9BP81 (**b**) were measured in 10 °C intervals from 25 °C to 95 °C. Both proteins retained their α-helical secondary structures at 95 °C, indicating high thermal stability. (**c**) A double phenylalanine to glutamate mutant of the designed retinal binding protein, RET2, named RET2_EE, was able to bind MeroCy7 and activate fluorescence in the far-red region. (**d**) Extinction coefficient determination for MC7BP34 (see supplement information). (**e**) Absolute photoluminescence quantum yield measurements for MC7BP34 protein solution (black) compared to a reference blank buffer (gray) when excited at 620 nm. (**f**) Mutating the designed lysine residue in the pocket to arginine completely eliminated dye binding, as evidenced by the disappearance of the 900 nm absorption peak in the mutant. The arginine mutant remains monomeric and folded (Figure S6).

**Figure S2.** Electrospray Ionization Mass Spectrometry of apo MC7BP34 (a and c) and the MC7BP34-MC7 complex (b and d). **(a)** and **(b)** The spectra with the mass range shown between 10,000 and 90,000 Da. **(c)** and **(d)** The spectra with the mass range shown between 40,000 and 50,000 Da. The major peak for apo MC7BP34 appears at 45,794 Da, corresponding to the intact protein lacking the N-terminal methionine after cleavage of the His tag, which is also present in the MC7BP34-MC7 complex sample. A distinct second peak is observed at 46,055 Da in the spectrum of the complex. The 261 Da mass shift is consistent with covalent MC7 labeling via Schiff base formation followed by sodium cyanoborohydride (50 mM) mediated reduction to a stable secondary amine.

**Figure S3.** Representative images of solutions and crystals of MeroCy7 and its complex with MC7BP34. (**a**) MeroCy7 dye solution in 0.2% n-Decyl-β-D-Maltopyranoside (left) and MC7BP34-MeroCy7 complex solution in PBS (right). The color difference reflects the distinctive absorption spectra of the dye and the protein-dye complex, as shown in Figure 2d. (**b**) Crystals formed by the MC7BP34-MeroCy7 complex. The dark blue color of the crystals suggests the presence of the dye covalently linked to the protein via a protonated Schiff base.

**Figure S4.** Comparison of our designed fluorescence-activating proteins with those cataloged in FPbase. (**a**) Maximum excitation and emission wavelengths for all fluorescent proteins in FPbase. Notably, the fluorescent protein iRFP720 and its derivative exhibit a maximum excitation wavelength of 702 nm and a maximum emission wavelength of 720 nm, representing the longest emission in the database. In contrast, MC9BP81 in complex with MeroCy9 demonstrates a redshift of approximately 200 nm compared to iRFP720. (**b**) The brightness of MC7BP34 in complex with MeroCy7 surpasses that of other fluorescent proteins with similar spectral profiles. Specifically, MC7BP34 achieves a brightness of 66.0, calculated as the product of the extinction coefficient and quantum yield divided by 1000, compared to a maximum brightness of 32.4 for others in this spectral window.

**Figure S5.** Dye labeling kinetics of MC7BP34. **(a)** The labeling kinetics were quantified by monitoring the time course of fluorescence intensity of MC7BP34-MC7 following MC7 dye addition using a microplate reader, with the MC7BP34 protein concentration fixed at 2 μM and varying concentrations of MC7 dye. Each dye concentration was measured in triplicate wells (n = 3). The fluorescence intensity time courses were well described by a single-exponential association model, from which observed rate constants (*k_obs_*) were extracted. **(b)** The dependence of *k_obs_* on MC7 concentration was analyzed using a linear relationship, *k_obs_* = *k_app_* [MC7] + k_0_, where *k_app_* corresponds to the apparent second-order labeling rate constant (slope) and *k_0_* is a concentration-independent offset (intercept).

**Figure S6.** Size-Exclusion Chromatography for MC7BP34, MC9BP81 and their knockout mutants. The lysine-to-arginine mutants elute at identical retention volumes as their original designs, indicating that the mutants remain monomeric and maintain proper folding.

**Figure S7.** Accumulation of MeroCy7 in cells. Untransfected U2OS cells were incubated with 0.3 μM MeroCy7 and imaged live by spinning disk confocal microscopy over time (single plane). Note the accumulation of MeroCy7 in intracellular compartments, requiring a pulse-chase labelling procedure when used for live cell imaging. The sample was excited using a 630 nm laser and imaged through a 647 nm long-pass filter. Scale bar: 10 μm.

**Figure S8.** Imaging contrast of MC7BP34 in live HEK293T cells. Time-lapse imaging was performed at 3-min intervals following addition of 0.3 μM MeroCy7 to the cells transfected with H2B-MC7BP34. Pixel intensities were normalized to the maximum value over the imaging period. The same image set is displayed using a high-intensity threshold **(a)** and a low-intensity threshold **(b)** to visualize the specific nuclear signal and background fluorescence, respectively. Scale bars, 10 μm.

**Figure S9.** Multicolor microscopy imaging in living HEK293T cells co-expressing SNAPf, HaloTag7 and MC7BP34. SNAPf was fused with tubulin and labeled with SNAP-Cell Oregon Green substrate. HaloTag7 was fused with the outer mitochondrial membrane protein Tomm20 and labeled with the JF549 HaloTag Ligand. MC7BP34 was fused with histone H2B and labeled with MeroCy7. Scale bars, 10 µm.

**Figure S10.** Electrospray Ionization Mass Spectrometry of apo MC9BP81 (a and c) and the MC9BP81-MC9 complex (b and d). **(a)** and **(b)** The spectra with the mass range shown between 10,000 and 90,000 Da. **(c)** and **(d)** The spectra with the mass range shown between 50,000 and 60,000 Da. The major peak for apo MC9BP81 appears at 53,802 Da, corresponding to the His-tag-cleaved protein lacking the N-terminal methionine, which is also present in the MC9BP81-MC9 complex sample. A distinct second peak is observed at 54,203 Da in the spectrum of the complex. The 401 Da mass shift is consistent with covalent MC9 labeling via Schiff base formation followed by sodium cyanoborohydride mediated reduction to a stable secondary amine.

**Figure S11.** Absorption spectra of chromophorylated MC9BP81 (10 µM) and ICG (2 µM).

**Figure S12.** SWIR imaging using MC9BP81. (**a**) Brightness comparison between ICG (green, excited at 785 nm) and MC9BP81-MeroCy9 complex (red, excited at 892 nm) as observed using different long-pass filters. ICG is approximately 15 times brighter than our designed NIR fluorescence-activating protein. (**b**) Absorption spectra of MC9BP81-EGFP-EGFRc and MC9BP81-EGFP-EGFRn showed that these constructs could be readily chromophorylated using MeroCy9. (**c**) Cell fixation did not affect the fluorescent signal. (**d**-**f**) Side-by-side comparison of labeled wild-type K562 cells and K562 cells expressing EGFR and iRFP using MC9BP81 fused with EGFR binding proteins. Following incubation with chromophorylated MC9BP81-EGFP-EGFRc and MC9BP81-EGFP-EGFRn proteins, K562 cells expressing EGFR and iRFP720 displayed a clear fluorescence signal when measured using a 900 nm longpass filter. In contrast, K562 cells not expressing EGFR showed no signal. (**e**) Zoomed-in image from (**d**) showed membrane localization after labeling the EGFR expressing K562 cells using purified MC9BP81-EGFP-EGFRc protein.

**Figure S13.** Macroscopic and in-vivo SWIR imaging using MC9BP81. (**a**) Alginate beads embedded with iRFP720 and EGFR expressing K562 cells showed SWIR fluorescence signal (950 nm longpass filter, excitation at 672 nm for iRFP720, 892 nm for MC9BP81). The error bars in the plot indicate the standard deviation of brightness across individual beads. (**b**) Despite the beads' higher emission at 672 nm excitation, imaging them at an excitation of 892 nm provides superior resolution and contrast, owing to the reduced tissue autofluorescence at longer wavelengths.

**Figure S14.** Cross-binding analysis of two designed protein-dye pairs. MC9BP81 was able to bind MeroCy7 at the tested concentration (10 µM protein, 5 µM dye; orange), likely due to MC9BP81's larger pocket, which can accommodate a smaller hydrophobic dye. However, this binding blue-shifts the absorption spectrum (orange curve, λ_max_ 480 nm; free dye λ_max_ 500 nm, see Figure 2) instead of shifting to the far-red region like the MC7BP34-MeroCy7 complex (blue curve, λ_max_ 675 nm), due to the absence of favorable local interactions. Therefore, this nonspecific binding does not cause optical interference to the MC7BP34-MeroCy7 system. MC7BP34, with its smaller binding pocket, does not interact with the bulkier MeroCy9 (red). These results demonstrate that our designed protein-dye systems can be used concurrently in imaging applications.

**Figure S15.** Dye labeling kinetics of MC9BP81. **(a)** The labeling kinetics were assessed by monitoring the time course of the absorption spectra of MC9BP81 at a fixed protein concentration of 10 μM and varying MC9 dye concentrations. **(b)** Reaction-associated spectral changes were quantified by numerical integration of baseline-corrected absorbance over the 880-920 nm wavelength window using the trapezoidal rule. Each dye concentration was measured in triplicate. Representative spectral profiles from these kinetic measurements are shown.

**Figure S16.** Binding competition experiments of MC7BP34 and MC9BP81. **(a)** MC7BP34 (2 μM) was preincubated for 30 min under the indicated buffer conditions (PBS; PBS supplemented with 10% fetal bovine serum (FBS); DMEM supplemented with 10% FBS; PBS with 4 μM retinal; or PBS with 4 μM retinol), followed by addition of MC7 (4 μM). Fluorescence signals were recorded using excitation at 640 ± 15 nm and emission at 690 ± 15 nm. The fluorescence intensity of the MC7–MC7BP34 complex remained stable over 48 h. Data are shown as mean fluorescence intensity ± SEM (n = 3 wells per condition) at selected time points. **(b)** MC9BP81 (10 μM) was preincubated for 30 min at room temperature under the indicated conditions (PBS; PBS supplemented with 10% FBS; DMEM supplemented with 10% FBS; PBS with 10 μM retinal; or PBS with 10 μM retinol). MC9 was then added to a final concentration of 10 μM to initiate labeling. Mean changes in near-infrared absorbance area (ΔAUC = AUC − AUC₀, 880–920 nm) are plotted as a function of time for MC9BP81 in PBS (blue), PBS supplemented with retinol (yellow), PBS supplemented with retinal (purple), PBS + 10% FBS (green), and DMEM + 10% FBS (red). A protein-only control (MC9BP81 without MC9; gray) is included to account for baseline drift. Data points represent the mean across replicate wells (n = 3), and error bars denote the standard error of the mean (SEM). Positive ΔAUC values indicate formation of the NIR-absorbing MC9BP81 complex, whereas negative values reflect suppression or loss of signal in serum-containing buffers.

### **Crystallography data**

**Table S1. Crystallographic statistics for WSHC5 and MC7BP34.**

|  | WSHC5 | MC7BP34 |
| --- | --- | --- |
| **Resolution range** | 40.67 - 3.05 (3.159 - 3.05) | 53.83 - 3.28 (3.40 - 3.28) |
| **Space group** | *P 2_1_ 2_1_ 2_1_* | *P 2_1_1* |
| **Unit cell** | 43.58, 74.35, 194.33; 90, 90, 90 | 54.19, 80.44, 72.63; 90, 94.26, 90 |
| **Unique reflections** | 11390 (1061) | 9537 (926) |
| **Multiplicity** | 5.7 (5.8) | 6.9 (7.2) |
| **Completeness (%)** | 90.17 (85.28) | 98.58 (96.56) |
| **Mean I/sigma(I)** | 7.86 (2.68) | 6.15 (0.82) |
| **Wilson B-factor** | 83.44 | 137.67 |
| **R-merge** | 0.1655 (0.5624) | 0.1716 (2.171) |
| **R-pim** | 0.07621 (0.2537) | 0.07115 (0.8674) |
| **CC1/2** | 0.968 (0.767) | 0.997 (0.634) |
| **Reflections used in refinement** | 11463 (1060) | 9482 (926) |
| **Reflections used for R-free** | 1174 (117) | 526 (41) |
| **R-work** | 0.2366 (0.2782) | 0.2641 (0.3729) |
| **R-free** | 0.2776 (0.3085) | 0.3206 (0.4530) |
| **Number of non-hydrogen atoms** | 4456 | 5509 |
| **macromolecules** | 4456 | 5509 |
| **ligands** | 0 | 0 |
| **solvent** | 0 | 0 |
| **Protein residues** | 670 | 647 |
| **RMS(bonds)** | 0.003 | 0.005 |
| **RMS(angles)** | 0.53 | 0.72 |
| **Ramachandran favored (%)** | 98.61 | 93.92 |
| **Ramachandran allowed (%)** | 1.39 | 5.42 |
| **Ramachandran outliers (%)** | 0.00 | 0.66 |
| **Average B-factor** | 60.76 | 147.97 |
| **macromolecules** | 60.76 | 147.97 |

Statistics for the highest-resolution shell are shown in parentheses.

### **Appendices**

**Appendix A.** Rosetta params files for MeroCy7-lysine NCAA

NAME MC7

IO_STRING MC7 X

AA UNK

TYPE POLYMER

LOWER_CONNECT N

UPPER_CONNECT C

PROPERTIES CANONICAL_AA ALPHA_AA L_AA

FIRST_SIDECHAIN_ATOM CB

ATOM N	Nbb NH1 0.42

ATOM CA CAbb CT1 0.13

ATOM C	CObb C	0.26

ATOM O	OCbb O	-0.28

ATOM CB CH2	X	0.00

ATOM CG CH2	X	-0.05

ATOM CD CH2	X	-0.04

ATOM CE CH2	X	0.01

ATOM NZ Ntrp X	-0.39

ATOM C20 aroC X	-0.00

ATOM C19 aroC X	-0.05

ATOM C18 aroC X	-0.06

ATOM C17 aroC X	-0.06

ATOM C16 aroC X	-0.06

ATOM C15 aroC X	-0.06

ATOM C14 aroC X	-0.06

ATOM C13 aroC X	-0.04

ATOM C8 aroC X	0.02

ATOM N9 Nhis X	-0.31

ATOM C12 CH3	X	0.01

ATOM H34 Hapo X	0.04

ATOM H33 Hapo X	0.04

ATOM H1 Hapo X	0.04

ATOM C3 aroC X	0.04

ATOM C4 aroC X	-0.04

ATOM C5 aroC X	-0.06

ATOM C6 aroC X	-0.06

ATOM C1 aroC X	-0.06

ATOM C2 aroC X	-0.01

ATOM C7 CH1	X	0.03

ATOM C11 CH3	X	-0.05

ATOM H29 Hapo X	0.02

ATOM H31 Hapo X	0.02

ATOM H30 Hapo X	0.02

ATOM C10 CH3	X	-0.05

ATOM H27 Hapo X	0.02

ATOM H28 Hapo X	0.02

ATOM H26 Hapo X	0.02

ATOM H22 Haro X	0.06

ATOM H25 Haro X	0.06

ATOM H24 Haro X	0.06

ATOM H23 Haro X	0.06

ATOM H35 Haro X	0.06

ATOM H36 Haro X	0.06

ATOM H37 Haro X	0.06

ATOM H38 Haro X	0.06

ATOM H39 Haro X	0.06

ATOM H2 Haro X	0.06

ATOM H40 Haro X	0.06

ATOM H41 Haro X	0.08

ATOM 1HZ Hpol X	1

ATOM 2HE Hapo X	0.05

ATOM 1HE Hapo X	0.05

ATOM 1HD Hapo X	0.03

ATOM 2HD Hapo X	0.03

ATOM 1HG Hapo X	0.03

ATOM 2HG Hapo X	0.03

ATOM 1HB Hapo X	0.03

ATOM 2HB Hapo X	0.03

ATOM HA Hapo HB	0.10

ATOM H	HNbb H	0.31

BOND_TYPE H34 C12 1

BOND_TYPE H33 C12 1

BOND_TYPE H23 C4 1

BOND_TYPE H1	C12 1

BOND_TYPE C12 N9 1

BOND_TYPE H24 C5 1

BOND_TYPE C4	C5 4

BOND_TYPE C4	C3 4

BOND_TYPE C5	C6 4

BOND_TYPE N9	C3 1

BOND_TYPE N9	C8 1

BOND_TYPE H35 C13 1

BOND_TYPE C3	C2 4

BOND_TYPE C8	C13 2

BOND_TYPE C8	C7 1

BOND_TYPE C13 C14 1

BOND_TYPE H37 C15 1

BOND_TYPE C6	H25 1

BOND_TYPE C6	C1 4

BOND_TYPE C2	C1 4

BOND_TYPE C2	C7 1

BOND_TYPE H29 C11 1

BOND_TYPE C1	H22 1

BOND_TYPE C15 C14 2

BOND_TYPE C15 C16 1

BOND_TYPE H39 C17 1

BOND_TYPE C14 H36 1

BOND_TYPE C7	C11 1

BOND_TYPE C7	C10 1

BOND_TYPE C11 H31 1

BOND_TYPE C11 H30 1

BOND_TYPE H40 C19 1

BOND_TYPE H27 C10 1

BOND_TYPE C17 C16 2

BOND_TYPE C17 C18 1

BOND_TYPE C16 H38 1

BOND_TYPE C10 H28 1

BOND_TYPE C10 H26 1

BOND_TYPE 1HZ NZ 1

BOND_TYPE C19 C18 2

BOND_TYPE C19 C20 1

BOND_TYPE 1HD CD 1

BOND_TYPE NZ	C20 2

BOND_TYPE NZ	CE 1

BOND_TYPE C20 H41 1

BOND_TYPE 1HG CG 1

BOND_TYPE CD	CE 1

BOND_TYPE CD	CG 1

BOND_TYPE CD	2HD 1

BOND_TYPE 2HG CG 1

BOND_TYPE 2HE CE 1

BOND_TYPE CE	1HE 1

BOND_TYPE CG	CB 1

BOND_TYPE O	C	2

BOND_TYPE HA	CA 1

BOND_TYPE CB	1HB 1

BOND_TYPE CB	CA 1

BOND_TYPE CB	2HB 1

BOND_TYPE C	CA 1

BOND_TYPE CA	N	1

BOND_TYPE N	H	1

BOND_TYPE C18 H2 1

CHI 1 N	CA	CB	CG

CHI 2 CA	CB	CG	CD

CHI 3 CB	CG	CD	CE

CHI 4 CG	CD	CE	NZ

CHI 5 CD	CE	NZ	C20

CHI 6 CE	NZ	C20 C19

CHARGE NZ FORMAL +1

NBR_ATOM CB

NBR_RADIUS 24.345934

ICOOR_INTERNAL	N	0.000000	0.000000	0.000000	N	CA	C

ICOOR_INTERNAL	CA	0.000000 180.000000	1.518980	N	CA	C

ICOOR_INTERNAL	C	0.000001	71.300159	1.557107	CA	N	C

ICOOR_INTERNAL	O	171.744902	62.333770	1.251953	C	CA	N

ICOOR_INTERNAL	CB -115.889612	71.425172	1.536202	CA	N	C

ICOOR_INTERNAL	CG -170.481454	66.096539	1.543178	CB	CA	N

ICOOR_INTERNAL	CD -129.193461	65.616421	1.538620	CG	CB	CA

ICOOR_INTERNAL	CE	68.311122	63.267860	1.532126	CD	CG	CB

ICOOR_INTERNAL	NZ	73.070579	68.623414	1.481097	CE	CD	CG

ICOOR_INTERNAL	C20 -150.831359	55.258268	1.286561	NZ	CE	CD

ICOOR_INTERNAL	C19 -179.248549	57.360775	1.477889	C20	NZ	CE

ICOOR_INTERNAL	C18 -170.712737	55.795245	1.348666	C19	C20	NZ

ICOOR_INTERNAL	C17 -177.453978	62.078123	1.466784	C18	C19	C20

ICOOR_INTERNAL	C16 176.652068	53.575613	1.342652	C17	C18	C19

ICOOR_INTERNAL	C15 -179.342773	57.267834	1.449432	C16	C17	C18

ICOOR_INTERNAL	C14 179.272076	57.180706	1.342200	C15	C16	C17

ICOOR_INTERNAL	C13 -179.824140	57.330165	1.448117	C14	C15	C16

ICOOR_INTERNAL	C8	177.968571	51.448175	1.349271	C13	C14	C15

ICOOR_INTERNAL	N9	179.206643	55.191149	1.407634	C8	C13	C14

ICOOR_INTERNAL	C12	8.110892	51.254349	1.450619	N9	C8	C13

ICOOR_INTERNAL	H34	93.783818	69.476203	1.095183	C12	N9	C8

ICOOR_INTERNAL	H33 121.231155	68.487648	1.095614	C12	N9	H34

ICOOR_INTERNAL	H1	118.314202	67.906809	1.093933	C12	N9	H33

ICOOR_INTERNAL	C3	167.799193	69.380522	1.399298	N9	C8	C12

ICOOR_INTERNAL	C4 -176.979506	50.529151	1.386924	C3	N9	C8

ICOOR_INTERNAL	C5 -179.389035	60.868928	1.398754	C4	C3	N9

ICOOR_INTERNAL	C6	-0.013471	59.285880	1.401330	C5	C4	C3

ICOOR_INTERNAL	C1	-0.163889	59.615371	1.394410	C6	C5	C4

ICOOR_INTERNAL	C2	0.037484	61.620870	1.390408	C1	C6	C5

ICOOR_INTERNAL	C7 -178.856279	50.912427	1.527702	C2	C1	C6

ICOOR_INTERNAL	C11	59.317756	71.331265	1.536584	C7	C2	C1

ICOOR_INTERNAL	H29	60.372440	68.555291	1.096431	C11	C7	C2

ICOOR_INTERNAL	H31 120.791624	67.552158	1.093031	C11	C7	H29

ICOOR_INTERNAL	H30 119.544900	68.596597	1.096807	C11	C7	H31

ICOOR_INTERNAL	C10 -122.140673	71.017352	1.535835	C7	C2	C11

ICOOR_INTERNAL	H27 -59.264281	68.539654	1.097510	C10	C7	C2

ICOOR_INTERNAL	H28 -120.522879	67.837515	1.094002	C10	C7	H27

ICOOR_INTERNAL	H26 -119.852468	68.615634	1.096158	C10	C7	H28

ICOOR_INTERNAL	H22 -179.796511	59.674657	1.084786	C1	C6	C2

ICOOR_INTERNAL	H25 -179.924419	60.242486	1.087460	C6	C5	C1

ICOOR_INTERNAL	H24 179.915339	60.293778	1.086839	C5	C4	C6

ICOOR_INTERNAL	H23 179.849618	58.386874	1.083355	C4	C3	C5

ICOOR_INTERNAL	H35 179.479598	66.187941	1.086625	C13	C14	C8

ICOOR_INTERNAL	H36 -179.657374	60.891472	1.083488	C14	C15	C13

ICOOR_INTERNAL	H37 -179.680736	62.695927	1.088728	C15	C16	C14

ICOOR_INTERNAL	H38 179.555762	58.813341	1.087570	C16	C17	C15

ICOOR_INTERNAL	H39 -179.326251	63.868588	1.090333	C17	C18	C16

ICOOR_INTERNAL	H2	179.999842	58.964031	1.032029	C18	C19	C17

ICOOR_INTERNAL	H40 -178.968416	63.777919	1.087155	C19	C20	C18

ICOOR_INTERNAL	H41 179.201978	62.006421	1.098829	C20	NZ	C19

ICOOR_INTERNAL	1HZ 176.478520	62.363745	1.020307	NZ	CE	C20

ICOOR_INTERNAL	2HE 116.268653	70.992461	1.094571	CE	CD	NZ

ICOOR_INTERNAL	1HE 120.183398	67.182130	1.093989	CE	CD	2HE

ICOOR_INTERNAL	1HD 123.829064	71.799764	1.098381	CD	CG	CE

ICOOR_INTERNAL	2HD 114.295078	72.056113	1.098059	CD	CG	1HD

ICOOR_INTERNAL	1HG 126.603122	67.620982	1.094599	CG	CB	CD

ICOOR_INTERNAL	2HG 114.776678	72.708085	1.097700	CG	CB	1HG

ICOOR_INTERNAL	1HB 120.011060	71.231489	1.098878	CB	CA	CG

ICOOR_INTERNAL	2HB 115.391192	70.048880	1.097708	CB	CA	1HB

ICOOR_INTERNAL	HA -122.151880	73.007665	1.092770	CA	N	CB

ICOOR_INTERNAL	H	138.048865	63.124655	1.020425	N	CA	C

ICOOR_INTERNAL UPPER 149.999985	63.800007	1.328685	C	CA	N

ICOOR_INTERNAL	O	-180.000000	59.200005	1.231015	C	CA UPPER

ICOOR_INTERNAL LOWER -150.000000	58.300003	1.328685	N	CA	C

ICOOR_INTERNAL	H	-180.000000	60.849998	1.010000	N	CA LOWER

ROTAMER_AA LYS

BACKBONE_AA LYS

**Appendix B.** Rosetta params file for MeroCy9-lysine NCAA

NAME MC9

IO_STRING MC9 X

AA UNK

TYPE POLYMER

LOWER_CONNECT N

UPPER_CONNECT C

PROPERTIES CANONICAL_AA ALPHA_AA L_AA SC_ORBITALS

FIRST_SIDECHAIN_ATOM CB

ATOM N	Nbb NH1 -0.25

ATOM CA CAbb CT1 0.09

ATOM C	CObb C	0.22

ATOM O	OCbb O	-0.29

ATOM CB CH2 CT2	-0.03

ATOM CG CH2 CT2	-0.05

ATOM CD CH2 CT2	0.01

ATOM CE CH2 CT2	0.14

ATOM NZ Ntrp X	-0.25

ATOM C16 aroC X	0.17

ATOM C15 aroC X	0.02

ATOM C14 aroC X	-0.00

ATOM C13 aroC X	-0.03

ATOM C12 aroC X	-0.01

ATOM C11 aroC X	-0.04

ATOM C10 aroC X	-0.06

ATOM C9 aroC X	-0.04

ATOM C8 aroC X	0.02

ATOM N1 Nhis X	-0.31

ATOM C1 aroC X	0.04

ATOM C2 aroC X	-0.04

ATOM C3 aroC X	-0.06

ATOM C4 aroC X	-0.06

ATOM C5 aroC X	-0.06

ATOM C6 aroC X	-0.01

ATOM C7 CH1	X	0.03

ATOM C18 CH3	X	-0.05

ATOM H14 Hapo X	0.02

ATOM H15 Hapo X	0.02

ATOM H16 Hapo X	0.02

ATOM C19 CH3	X	-0.05

ATOM H11 Hapo X	0.02

ATOM H12 Hapo X	0.02

ATOM H13 Hapo X	0.02

ATOM H4 Haro X	0.06

ATOM H3 Haro X	0.06

ATOM H2 Haro X	0.06

ATOM H1 Haro X	0.06

ATOM C17 CH3	X	0.01

ATOM H8 Hapo X	0.04

ATOM H9 Hapo X	0.04

ATOM H10 Hapo X	0.04

ATOM H5 Haro X	0.06

ATOM H6 Haro X	0.06

ATOM C20 CH2	X	-0.03

ATOM C21 CH2	X	-0.04

ATOM C22 CH1	X	-0.01

ATOM C23 CH2	X	-0.04

ATOM C24 CH2	X	-0.02

ATOM H24 Hapo X	0.03

ATOM H25 Hapo X	0.03

ATOM H22 Hapo X	0.03

ATOM H23 Hapo X	0.03

ATOM H21 Hapo X	0.03

ATOM H19 Hapo X	0.03

ATOM H20 Hapo X	0.03

ATOM H17 Hapo X	0.03

ATOM H18 Hapo X	0.03

ATOM C25 aroC X	-0.01

ATOM C26 aroC X	-0.05

ATOM C27 aroC X	-0.06

ATOM C28 aroC X	-0.06

ATOM C29 aroC X	-0.05

ATOM C30 aroC X	-0.01

ATOM H29 Haro X	0.06

ATOM H28 Haro X	0.06

ATOM H27 Haro X	0.06

ATOM H26 Haro X	0.06

ATOM H7 Haro X	0.14

ATOM HZ Hpol X	0.45

ATOM 1HE Hapo X	0.10

ATOM 2HE Hapo X	0.10

ATOM 1HD Hapo X	0.03

ATOM 2HD Hapo X	0.03

ATOM 1HG Hapo X	0.03

ATOM 2HG Hapo X	0.03

ATOM 1HB Hapo X	0.03

ATOM 2HB Hapo X	0.03

ATOM HA Hapo HB	0.06

ATOM H	HNbb H	0.14

BOND_TYPE N	CA 1

BOND_TYPE N	H	1

BOND_TYPE CA	C	1

BOND_TYPE CA	CB 1

BOND_TYPE CA	HA 1

BOND_TYPE C	O	2

BOND_TYPE CB	CG 1

BOND_TYPE CB	1HB 1

BOND_TYPE CB	2HB 1

BOND_TYPE CG	CD 1

BOND_TYPE CG	1HG 1

BOND_TYPE CG	2HG 1

BOND_TYPE CD	CE 1

BOND_TYPE CD	1HD 1

BOND_TYPE CD	2HD 1

BOND_TYPE CE	NZ 1

BOND_TYPE CE	1HE 1

BOND_TYPE CE	2HE 1

BOND_TYPE NZ	C16 2

BOND_TYPE NZ	HZ 1

BOND_TYPE C1	N1 1

BOND_TYPE C1	C2 4

BOND_TYPE C1	C6 4

BOND_TYPE N1	C8 1

BOND_TYPE N1	C17 1

BOND_TYPE C2	C3 4

BOND_TYPE C2	H1 1

BOND_TYPE C3	C4 4

BOND_TYPE C3	H2 1

BOND_TYPE C4	C5 4

BOND_TYPE C4	H3 1

BOND_TYPE C5	C6 4

BOND_TYPE C5	H4 1

BOND_TYPE C6	C7 1

BOND_TYPE C7	C8 1

BOND_TYPE C7	C18 1

BOND_TYPE C7	C19 1

BOND_TYPE C8	C9 2

BOND_TYPE C9	C10 1

BOND_TYPE C9	H5 1

BOND_TYPE C10 C11 2

BOND_TYPE C10 H6 1

BOND_TYPE C11 C12 1

BOND_TYPE C11 C20 1

BOND_TYPE C12 C13 2

BOND_TYPE C12 C25 1

BOND_TYPE C13 C14 1

BOND_TYPE C13 C22 1

BOND_TYPE C14 C15 2

BOND_TYPE C14 C30 1

BOND_TYPE C15 C16 1

BOND_TYPE C15 C24 1

BOND_TYPE C16 H7 1

BOND_TYPE C17 H8 1

BOND_TYPE C17 H9 1

BOND_TYPE C17 H10 1

BOND_TYPE C18 H14 1

BOND_TYPE C18 H15 1

BOND_TYPE C18 H16 1

BOND_TYPE C19 H11 1

BOND_TYPE C19 H12 1

BOND_TYPE C19 H13 1

BOND_TYPE C20 C21 1

BOND_TYPE C20 H17 1

BOND_TYPE C20 H18 1

BOND_TYPE C21 C22 1

BOND_TYPE C21 H19 1

BOND_TYPE C21 H20 1

BOND_TYPE C22 C23 1

BOND_TYPE C22 H21 1

BOND_TYPE C23 C24 1

BOND_TYPE C23 H22 1

BOND_TYPE C23 H23 1

BOND_TYPE C24 H24 1

BOND_TYPE C24 H25 1

BOND_TYPE C25 C26 4

BOND_TYPE C25 C30 4

BOND_TYPE C26 C27 4

BOND_TYPE C26 H26 1

BOND_TYPE C27 C28 4

BOND_TYPE C27 H27 1

BOND_TYPE C28 C29 4

BOND_TYPE C28 H28 1

BOND_TYPE C29 C30 4

BOND_TYPE C29 H29 1

CHI 1 N	CA	CB	CG

CHI 2 CA	CB	CG	CD

CHI 3 CB	CG	CD	CE

CHI 4 CG	CD	CE	NZ

CHI 5 CD	CE	NZ	C16

CHI 6 CE	NZ	C16 C15

CHARGE NZ FORMAL +1

NBR_ATOM CB

NBR_RADIUS 23.464090

ICOOR_INTERNAL	N	0.000000	0.000000	0.000000	N	CA	C

ICOOR_INTERNAL	CA	0.000000 180.000000	1.471134	N	CA	C

ICOOR_INTERNAL	C	0.000001	71.700993	1.554704	CA	N	C

ICOOR_INTERNAL	O	-15.087048	67.662123	1.336681	C	CA	N

ICOOR_INTERNAL	CB -119.094385	68.972889	1.544793	CA	N	C

ICOOR_INTERNAL	CG -170.720890	66.955972	1.532448	CB	CA	N

ICOOR_INTERNAL	CD -172.919397	66.676023	1.531892	CG	CB	CA

ICOOR_INTERNAL	CE	178.372124	68.276262	1.529752	CD	CG	CB

ICOOR_INTERNAL	NZ -176.173178	68.118793	1.461208	CE	CD	CG

ICOOR_INTERNAL	C16 150.276111	55.133821	1.344780	NZ	CE	CD

ICOOR_INTERNAL	C15 -177.617305	54.040411	1.396623	C16	NZ	CE

ICOOR_INTERNAL	C14 -174.223872	57.583193	1.422140	C15	C16	NZ

ICOOR_INTERNAL	C13 -166.469894	59.865154	1.409898	C14	C15	C16

ICOOR_INTERNAL	C12 -167.381904	68.969952	1.419981	C13	C14	C15

ICOOR_INTERNAL	C11 172.038767	59.597302	1.417263	C12	C13	C14

ICOOR_INTERNAL	C10 176.370579	58.052775	1.407828	C11	C12	C13

ICOOR_INTERNAL	C9 -179.049290	53.949892	1.406131	C10	C11	C12

ICOOR_INTERNAL	C8	179.927087	55.071056	1.401150	C9	C10	C11

ICOOR_INTERNAL	N1 -179.856939	57.702667	1.367533	C8	C9	C10

ICOOR_INTERNAL	C1 -179.005180	68.369974	1.413983	N1	C8	C9

ICOOR_INTERNAL	C2	179.662726	50.886318	1.401726	C1	N1	C8

ICOOR_INTERNAL	C3	179.960759	62.755698	1.406824	C2	C1	N1

ICOOR_INTERNAL	C4	-0.040381	58.779298	1.407171	C3	C2	C1

ICOOR_INTERNAL	C5	0.094220	59.440652	1.410076	C4	C3	C2

ICOOR_INTERNAL	C6	-0.037959	61.187172	1.395447	C5	C4	C3

ICOOR_INTERNAL	C7	179.937297	49.225970	1.522714	C6	C5	C4

ICOOR_INTERNAL	C18	61.809369	69.878743	1.548974	C7	C6	C5

ICOOR_INTERNAL	H14	58.735979	69.012397	1.107380	C18	C7	C6

ICOOR_INTERNAL	H15 -119.166644	70.466995	1.107912	C18	C7	H14

ICOOR_INTERNAL	H16 -119.857805	68.113597	1.106950	C18	C7	H15

ICOOR_INTERNAL	C19 -123.606659	69.982274	1.548274	C7	C6	C18

ICOOR_INTERNAL	H11 -56.125153	68.958766	1.106837	C19	C7	C6

ICOOR_INTERNAL	H12 -120.837336	68.041283	1.107638	C19	C7	H11

ICOOR_INTERNAL	H13 -119.950976	70.389871	1.108349	C19	C7	H12

ICOOR_INTERNAL	H4 -179.941466	59.730215	1.101159	C5	C4	C6

ICOOR_INTERNAL	H3	179.987523	60.314977	1.098964	C4	C3	C5

ICOOR_INTERNAL	H2 -179.904594	60.881239	1.099915	C3	C2	C4

ICOOR_INTERNAL	H1	179.922632	57.666997	1.099200	C2	C1	C3

ICOOR_INTERNAL	C17 179.703853	55.843082	1.453564	N1	C8	C1

ICOOR_INTERNAL	H8	177.795621	70.444285	1.102308	C17	N1	C8

ICOOR_INTERNAL	H9 -119.439670	69.515897	1.109094	C17	N1	H8

ICOOR_INTERNAL	H10 -120.781555	69.313081	1.108447	C17	N1	H9

ICOOR_INTERNAL	H5	179.992639	62.013331	1.099357	C9	C10	C8

ICOOR_INTERNAL	H6	178.711451	63.462446	1.093423	C10	C11	C9

ICOOR_INTERNAL	C20 -179.589599	62.273389	1.526864	C11	C12	C10

ICOOR_INTERNAL	C21 -18.762741	64.800073	1.539543	C20	C11	C12

ICOOR_INTERNAL	C22	50.129025	70.376409	1.540564	C21	C20	C11

ICOOR_INTERNAL	C23 175.419497	62.389758	1.552615	C22	C21	C20

ICOOR_INTERNAL	C24 105.242344	65.425408	1.543546	C23	C22	C21

ICOOR_INTERNAL	H24 -73.313181	70.512602	1.113867	C24	C23	C22

ICOOR_INTERNAL	H25 -115.129092	71.829574	1.110195	C24	C23	H24

ICOOR_INTERNAL	H22 120.735639	71.426388	1.108817	C23	C22	C24

ICOOR_INTERNAL	H23 115.772759	70.079624	1.108689	C23	C22	H22

ICOOR_INTERNAL	H21 -118.807306	74.053038	1.115696	C22	C21	C23

ICOOR_INTERNAL	H19 -120.636966	69.748997	1.111916	C21	C20	C22

ICOOR_INTERNAL	H20 -117.660759	70.635331	1.110028	C21	C20	H19

ICOOR_INTERNAL	H17 -123.412852	70.835614	1.110420	C20	C11	C21

ICOOR_INTERNAL	H18 -114.520712	71.688176	1.115090	C20	C11	H17

ICOOR_INTERNAL	C25 -176.625814	73.251145	1.495479	C12	C13	C11

ICOOR_INTERNAL	C26 -179.181902	46.524415	1.398664	C25	C12	C13

ICOOR_INTERNAL	C27 -178.248963	60.131020	1.413468	C26	C25	C12

ICOOR_INTERNAL	C28	1.455957	59.371291	1.398794	C27	C26	C25

ICOOR_INTERNAL	C29	-0.480855	59.781492	1.413278	C28	C27	C26

ICOOR_INTERNAL	C30	-1.660459	60.479313	1.397594	C29	C28	C27

ICOOR_INTERNAL	H29 179.165476	60.935778	1.097842	C29	C28	C30

ICOOR_INTERNAL	H28 179.523981	59.908263	1.099343	C28	C27	C29

ICOOR_INTERNAL	H27 179.343931	60.574749	1.100444	C27	C26	C28

ICOOR_INTERNAL	H26 179.705999	57.929804	1.097868	C26	C25	C27

ICOOR_INTERNAL	H7	177.056307	65.518067	1.099999	C16	NZ	C15

ICOOR_INTERNAL	HZ	176.807448	63.067736	1.024820	NZ	CE	C16

ICOOR_INTERNAL	1HE 120.108924	70.134522	1.109321	CE	CD	NZ

ICOOR_INTERNAL	2HE 117.375964	69.976058	1.114228	CE	CD	1HE

ICOOR_INTERNAL	1HD -121.024966	70.243541	1.112024	CD	CG	CE

ICOOR_INTERNAL	2HD -117.119849	69.872224	1.114695	CD	CG	1HD

ICOOR_INTERNAL	1HG 122.416396	71.517401	1.110437	CG	CB	CD

ICOOR_INTERNAL	2HG 114.588824	70.726542	1.112315	CG	CB	1HG

ICOOR_INTERNAL	1HB -122.487577	71.198558	1.113090	CB	CA	CG

ICOOR_INTERNAL	2HB -115.327425	71.548662	1.113580	CB	CA	1HB

ICOOR_INTERNAL	HA -121.741805	66.704272	1.112254	CA	N	CB

ICOOR_INTERNAL	H	-94.566275	67.795603	1.026581	N	CA	C

ICOOR_INTERNAL UPPER 149.999985	63.800007	1.328685	C	CA	N

ICOOR_INTERNAL	O	-180.000000	59.200005	1.231015	C	CA UPPER

ICOOR_INTERNAL LOWER -150.000000	58.300003	1.328685	N	CA	C

ICOOR_INTERNAL	H	-180.000000	60.849998	1.010000	N	CA LOWER

ROTAMER_AA LYS

BACKBONE_AA LYS

**Appendix C.** Example RosettaScripts XML protocol for design of ligand binding proteins.

<ROSETTASCRIPTS>

<SCOREFXNS>

<ScoreFunction name="soft" weights="beta_soft.wts" symmetric="0">

<Reweight scoretype="aa_composition" weight="1.0" />

</ScoreFunction>

<ScoreFunction name="hard" weights="beta_cst" symmetric="0">

<Reweight scoretype="coordinate_constraint" weight="0.5" />

<Reweight scoretype="aa_composition" weight="1.0" />

<Reweight scoretype="angle_constraint" weight="3" />

<Reweight scoretype="dihedral_constraint" weight="3" />

</ScoreFunction>

<ScoreFunction name="hard_cart" weights="beta_cart" symmetric="0" >

<Reweight scoretype="cart_bonded" weight="0.5" />

<Reweight scoretype="coordinate_constraint" weight="1" />

<Reweight scoretype="angle_constraint" weight="3" />

<Reweight scoretype="dihedral_constraint" weight="3" />

</ScoreFunction>

<ScoreFunction name="hard_ele" weights="beta_cst" symmetric="0">

<Reweight scoretype="aa_composition" weight="1.0" />

<Reweight scoretype="fa_elec" weight="1.4" />

<Reweight scoretype="hbond_sc" weight="2.0" />

<Reweight scoretype="angle_constraint" weight="3" />

<Reweight scoretype="dihedral_constraint" weight="3" />

</ScoreFunction>

<ScoreFunction name="output" weights="beta" symmetric="0">

<Reweight scoretype="cart_bonded" weight="0.5" />

<Reweight scoretype="coordinate_constraint" weight="1" />

<Reweight scoretype="angle_constraint" weight="3" />

<Reweight scoretype="dihedral_constraint" weight="3" />

</ScoreFunction>

<ScoreFunction name="beta" weights="beta" symmetric="0" />

</SCOREFXNS>

<PACKER_PALETTES>

<CustomBaseTypePackerPalette name="custom_palette" additional_residue_types="MC9"/>

</PACKER_PALETTES>

<RESIDUE_SELECTORS>

<Index name="MC9_index" resnums="51A" />

<Index name="pore" resnums="44A,47A,51A,54A,58A,61A,65A,124A,127A,131A,134A,138A,141A,145A,203A,206A,210A,213A,217A,220A,224A,282A,285A,289A,292A,296A,299A,303A,361A,364A,368A,371A,375A,378A,382A,440A,443A,447A,450A,454A,457A,461A" />

<Index name="loop" resnums="71A,72A,73A,74A,75A,76A,77A,78A,79A,80A,81A,150A,151A,152A,153A,154A,155A,156A,157A,158A,159A,160A,229A,230A,231A,232A,233A,234A,235A,236A,237A,238A,239A,308A,309A,310A,311A,312A,313A,314A,315A,316A,317A,318A,387A,388A,389A,390A,391A,392A,393A,394A,395A,396A,397A" />

<Not name="not_pore" selector="pore" />

<Not name="not_MC9" selector="MC9_index" />

<Neighborhood name="MC9_neighbor" selector="MC9_index" distance="10" />

<ResidueName name="select_pro_gly" residue_name3="PRO,GLY" />

<SecondaryStructure name="select_loop" ss="L" include_terminal_loops="false" use_dssp="true" overlap="1" />

<And name="select_pro_gly_in_loop" selectors="select_loop,select_pro_gly" />

<CloseContact name="MC9_interface" residue_selector="MC9_index" contact_threshold="6" />

<CloseContact name="MC9_interface_loose" residue_selector="MC9_index" contact_threshold="8" />

<And name="MC9_interface_with_pore" selectors="MC9_interface,pore"/>

<And name="MC9_interface_loose_with_pore" selectors="MC9_interface_loose,pore"/>

<Or name="MC9_interface_with_pore_plus_loop" selectors="loop,MC9_interface_with_pore" />

<Or name="MC9_interface_loose_with_pore_plus_loop" selectors="loop,MC9_interface_loose_with_pore" />

<Not name="not_MC9_interface" selector="MC9_interface_with_pore_plus_loop" />

<Not name="not_MC9_interface_loose" selector="MC9_interface_loose_with_pore_plus_loop" />

<ResiduePDBInfoHasLabel name="hbnet_residues" property="HBNet" />

<Neighborhood name="around_hbnet" selector="hbnet_residues" distance="5.0" />

<Not name="not_around_hbnet" selector="around_hbnet" />

<Layer name="hbnet_core" select_core="true" core_cutoff="3.6" />

<And name="core_around_hbnet" selectors="hbnet_core,around_hbnet"/>

<Layer name="pick_core_and_boundary" select_core="true" select_boundary="true" core_cutoff="3.6"/>

<Layer name="pick_core_and_boundary_SASA" use_sidechain_neighbors="false" select_core="true" select_boundary="true" core_cutoff="35" surface_cutoff="45" />

<Layer name="pick_core_and_surface" select_core="true" select_surface="true" core_cutoff="3.6"/>

<Layer name="pick_core_and_surface_SASA" use_sidechain_neighbors="false" select_core="true" select_surface="true" core_cutoff="35" surface_cutoff="45"/>

<Layer name="pick_surface_and_boundary" select_surface="true" select_boundary="true" core_cutoff="3.6"/>

<Layer name="pick_surface_and_boundary_SASA" use_sidechain_neighbors="false" select_surface="true" select_boundary="true" core_cutoff="35" surface_cutoff="45" />

</RESIDUE_SELECTORS>

<TASKOPERATIONS>

<InitializeFromCommandline name="init"/>

<IncludeCurrent name="current"/>

<LimitAromaChi2 name="arochi" />

<ExtraRotamersGeneric name="ex1_ex2" ex1="1" ex2="1" ex1_sample_level="3" ex2_sample_level="3" />

<ExtraRotamersGeneric name="ex1" ex1="1" ex1_sample_level="3"/>

<ReadResfile name="resfile" filename="%%resfile%%" />

ReadResfile name="hbnet_resfile" filename="hbnet.resfile"/>

<LayerDesign name="init_layers" layer="core_boundary_surface_Nterm_Cterm" ignore_pikaa_natro="true" make_pymol_script="0" use_sidechain_neighbors="True" core="3.6" >

<core>

<Helix append="M,W,Y,F,MC9"/>

</core>

<boundary>

<Helix append="M,W,Y,F,MC9"/>

<Helix exclude="C,D,R,H,Q"/>

</boundary>

<surface>

<Helix exclude="CDHNQSTR"/>

</surface>

</LayerDesign>

<LayerDesign name="sasa_layers" layer="core_boundary_surface_Nterm_Cterm" ignore_pikaa_natro="true" make_pymol_script="0" use_sidechain_neighbors="0" core="35" surface="45" >

<core>

<Helix append="M,W,Y,F,MC9"/>

</core>

<boundary>

<Helix append="M,W,Y,F,MC9"/>

<Helix exclude="CDRH"/>

</boundary>

<surface>

<Helix exclude="CDST"/>

</surface>

</LayerDesign>

<SelectBySASA name="select_core" state="bound" mode="mc" core="1" probe_radius="2.0" core_asa="35" surface_asa="45" verbose="1"/>

<SelectBySASA name="select_boundary" state="bound" mode="mc" boundary="1" probe_radius="2.0" core_asa="35" surface_asa="45" verbose="1"/>

<SelectBySASA name="select_surface" state="bound" mode="mc" surface="1" probe_radius="2.0" core_asa="35" surface_asa="45" verbose="1"/>

<RestrictToRepacking name="repack_only" />

<ConsensusLoopDesign name="disallow_non_abego_aas"/>

<OperateOnResidueSubset name="hbnet_task" selector="hbnet_residues">

<RestrictToRepackingRLT/>

</OperateOnResidueSubset>

<OperateOnResidueSubset name="hbnet_task_no_repack" selector="hbnet_residues">

<PreventRepackingRLT/>

</OperateOnResidueSubset>

<OperateOnResidueSubset name="repack_only_if_not_around_hbnet" selector="not_around_hbnet">

<RestrictToRepackingRLT/>

</OperateOnResidueSubset>

<OperateOnResidueSubset name="pro_gly_repack_only" selector="select_pro_gly_in_loop" >

<RestrictToRepackingRLT/>

</OperateOnResidueSubset>

<OperateOnResidueSubset name="design_core" selector="pick_surface_and_boundary">

<PreventRepackingRLT/>

</OperateOnResidueSubset>

<OperateOnResidueSubset name="design_boundary" selector="pick_core_and_surface">

<PreventRepackingRLT/>

</OperateOnResidueSubset>

<OperateOnResidueSubset name="design_surface" selector="pick_core_and_boundary">

<PreventRepackingRLT/>

</OperateOnResidueSubset>

<OperateOnResidueSubset name="design_core_SASA" selector="pick_surface_and_boundary_SASA">

<PreventRepackingRLT/>

</OperateOnResidueSubset>

<OperateOnResidueSubset name="design_boundary_SASA" selector="pick_core_and_surface_SASA">

<PreventRepackingRLT/>

</OperateOnResidueSubset>

<OperateOnResidueSubset name="design_surface_SASA" selector="pick_core_and_boundary_SASA">

<PreventRepackingRLT/>

</OperateOnResidueSubset>

<OperateOnResidueSubset name="repack_only_if_not_MC9" selector="not_MC9" >

<RestrictToRepackingRLT/>

</OperateOnResidueSubset>

<OperateOnResidueSubset name="no_repack_if_not_MC9" selector="not_MC9" >

<PreventRepackingRLT/>

</OperateOnResidueSubset>

<OperateOnResidueSubset name="repack_only_if_not_MC9_interface" selector="not_MC9_interface" >

<RestrictToRepackingRLT/>

</OperateOnResidueSubset>

<OperateOnResidueSubset name="no_repack_if_not_MC9_interface_loose" selector="not_MC9_interface_loose" >

<PreventRepackingRLT/>

</OperateOnResidueSubset>

</TASKOPERATIONS>

<FILTERS>

<ShapeComplementarity name="sc" min_sc="0.0" verbose="1" write_int_area="1" residue_selector1="MC9_index" residue_selector2="not_MC9" />

<TotalSasa name="sasa_MC9" threshold="0" upper_threshold="100" hydrophobic="0" polar="0" task_operations="no_repack_if_not_MC9" confidence="0" />

<TaskAwareScoreType name="score_MC9" task_operations="no_repack_if_not_MC9" scorefxn="beta" score_type="total_score" threshold="0" mode="total" unbound="0" />

<ResidueCount name="tyr_count" residue_types="TYR" confidence="0" />

<ResidueCount name="phe_count" residue_types="PHE" confidence="0" />

<ResidueCount name="trp_count" residue_types="TRP" confidence="0" />

<CombinedValue name="aromatic_count" threshold="50" confidence="0">

<Add filter_name="tyr_count" factor="1.0"/>

<Add filter_name="phe_count" factor="1.0"/>

<Add filter_name="trp_count" factor="1.0"/>

</CombinedValue>

<SSPrediction name="ss_pred" use_svm="0" use_probability="1" cmd="/home/cxu7/bin/psipred/runpsipred_single" />

<SSPrediction name="ss_pred_percentage" threshold="0.90" use_probability="0" use_svm="0" cmd="/home/cxu7/bin/psipred/runpsipred_single" />

<ScoreType name="total_score" scorefxn="hard" score_type="total_score" threshold="0" confidence="0" />

<ResidueCount name="ala_count" max_residue_count="30" residue_types="ALA" confidence="0"/>

<PreProline name="prepro" use_statistical_potential="0" />

</FILTERS>

<MOVERS>

<AddConstraintsToCurrentConformationMover name="add_cst" use_distance_cst="0" coord_dev="0.5" />

<AddCompositionConstraintMover name="addcomp" filename="%%aa_comp%%" selector="MC9_interface" />

<ConstraintSetMover name="add_dihedral_cst" add_constraints="1" cst_file="%%dihedral_cst%%"/>

<PackRotamersMover name="softpack_all" scorefxn="soft" task_operations="init,init_layers,current,arochi,hbnet_task_no_repack,disallow_non_abego_aas"/>

<PackRotamersMover name="softpack_core_init" packer_palette="custom_palette" scorefxn="soft" task_operations="resfile,init,init_layers,current,design_core_SASA,disallow_non_abego_aas,ex1_ex2,pro_gly_repack_only,no_repack_if_not_MC9" />

<PackRotamersMover name="softpack_core" packer_palette="custom_palette" scorefxn="soft" task_operations="init,resfile,sasa_layers,current,design_core_SASA,arochi,hbnet_task_no_repack,disallow_non_abego_aas,pro_gly_repack_only,repack_only_if_not_MC9_interface,no_repack_if_not_MC9_interface_loose" />

<PackRotamersMover name="softpack_boundary" packer_palette="custom_palette" scorefxn="soft" task_operations="init,resfile,sasa_layers,current,design_boundary_SASA,arochi,hbnet_task_no_repack,disallow_non_abego_aas,pro_gly_repack_only,repack_only_if_not_MC9_interface,no_repack_if_not_MC9_interface_loose" />

<PackRotamersMover name="softpack_surface" packer_palette="custom_palette" scorefxn="soft" task_operations="init,resfile,sasa_layers,current,design_surface_SASA,arochi,hbnet_task_no_repack,disallow_non_abego_aas,pro_gly_repack_only,repack_only_if_not_MC9_interface,no_repack_if_not_MC9_interface_loose" />

<PackRotamersMover name="hardpack_core" packer_palette="custom_palette" scorefxn="hard" task_operations="init,resfile,sasa_layers,current,design_core_SASA,arochi,ex1_ex2,hbnet_task_no_repack,disallow_non_abego_aas,pro_gly_repack_only,repack_only_if_not_MC9_interface,no_repack_if_not_MC9_interface_loose" />

<PackRotamersMover name="hardpack_boundary" packer_palette="custom_palette" scorefxn="hard" task_operations="init,resfile,sasa_layers,current,design_boundary_SASA,arochi,ex1,hbnet_task_no_repack,disallow_non_abego_aas,pro_gly_repack_only,repack_only_if_not_MC9_interface,no_repack_if_not_MC9_interface_loose" />

<PackRotamersMover name="hardpack_surface" packer_palette="custom_palette" scorefxn="hard_ele" task_operations="init,resfile,sasa_layers,current,design_surface_SASA,arochi,hbnet_task_no_repack,disallow_non_abego_aas,pro_gly_repack_only,repack_only_if_not_MC9_interface,no_repack_if_not_MC9_interface_loose" />

<PackRotamersMover name="hardpack_all" packer_palette="custom_palette" scorefxn="hard" task_operations="init,resfile,sasa_layers,current,arochi,hbnet_task_no_repack,disallow_non_abego_aas,pro_gly_repack_only,repack_only_if_not_MC9_interface,no_repack_if_not_MC9_interface_loose" />

<MinMover name="hardmin_sconly" scorefxn="hard" type="lbfgs_armijo_nonmonotone" chi="1" bb="0" bondangle="0" bondlength="0" />

<MinMover name="hardmin_cart" scorefxn="hard_cart" type="lbfgs_armijo_nonmonotone" tolerance="0.0001" max_iter="2000" chi="1" bb="1" bondangle="1" bondlength="1" jump="ALL" cartesian="1"/>

<PackRotamersMover name="repack" packer_palette="custom_palette" scorefxn="hard" task_operations="init,repack_only,ex1_ex2,no_repack_if_not_MC9_interface_loose" />

<InterfaceAnalyzerMover name="interface_analyzer" scorefxn="hard" packstat="1" pack_input="0" pack_separated="1" interface_sc="1"/>

<ParsedProtocol name="pack_min_core">

<Add mover_name="softpack_core"/>

<Add mover_name="softpack_boundary"/>

<Add mover_name="softpack_surface"/>

Add mover_name="hardmin_sconly"/>

<Add mover_name="hardmin_cart"/>

<Add mover_name="hardpack_core"/>

<Add mover_name="hardpack_boundary"/>

<Add mover_name="hardpack_surface"/>

</ParsedProtocol>

<GenericSimulatedAnnealer name="GSA_pack_min_core"

mover_name="pack_min_core" trials="10"

periodic_mover="hardpack_all" eval_period="20" history="10"

bolz_rank="1" recover_low="1" preapply="0" drift="1"

filter_name="sc" temperature="0.001" sample_type="high" >

<Filters>

<AND filter_name="sasa_MC9" temperature="0.5" sample_type="low"/>

</Filters>

</GenericSimulatedAnnealer> #stopping_condition="ss_pred_percentage"

</MOVERS>

<APPLY_TO_POSE>

</APPLY_TO_POSE>

<PROTOCOLS>

<Add mover="add_cst"/>

<Add mover="hardmin_cart" />

<Add mover="softpack_core_init"/>

<Add mover="addcomp"/>

<Add mover="add_dihedral_cst"/>

<Add mover="GSA_pack_min_core"/>

<Add mover="hardmin_cart" />

<Add mover="repack" />

<Add filter="aromatic_count"/>

<Add filter="score_MC9"/>

<Add filter="sasa_MC9"/>

<Add filter="sc"/>

</PROTOCOLS>

<OUTPUT scorefxn="output" />

</ROSETTASCRIPTS>

**Appendix D.** Example amino acid constraint file for use in the above RosettaScripts XML protocol:

PENALTY_DEFINITION

PROPERTIES AROMATIC

ABSOLUTE 7

PENALTIES 2000 1000 0 0 0

DELTA_START -2

DELTA_END 2

BEFORE_FUNCTION LINEAR

AFTER_FUNCTION CONSTANT

END_PENALTY_DEFINITION

PENALTY_DEFINITION

TYP ALA

ABSOLUTE 3

PENALTIES 0 0 0 5000 10000

DELTA_START -2

DELTA_END 2

BEFORE_FUNCTION CONSTANT

AFTER_FUNCTION LINEAR

END_PENALTY_DEFINITION

**Appendix E.** Example resfile for use in the above RosettaScripts XML protocol:

NOTAA X[MC9]

start

51 A PIKAA X[MC9]

**Appendix F.** Example geometry constraint file for use in the above RosettaScripts XML protocol:

Dihedral C15 51 C16 51 NZ 51 CE 51 CIRCULARHARMONIC 3.18317853263 0.2

Angle C15 51 C16 51 NZ 51 HARMONIC 2.19840954415 0.2

Angle C16 51 NZ 51 CE 51 HARMONIC 2.17932595278 0.2

Dihedral C14 51 C15 51 C16 51 NZ 51 CIRCULARHARMONIC 3.24240510303 0.2

Angle C14 51 C15 51 C16 51 HARMONIC 2.13657633459 0.2

Angle C15 51 C16 51 NZ 51 HARMONIC 2.19840954415 0.2

Dihedral C13 51 C14 51 C15 51 C16 51 CIRCULARHARMONIC 3.37773754759 0.2

Angle C13 51 C14 51 C15 51 HARMONIC 2.09674861599 0.2

Angle C14 51 C15 51 C16 51 HARMONIC 2.13657633459 0.2

Dihedral C12 51 C13 51 C14 51 C15 51 CIRCULARHARMONIC 3.3618199778 0.2

Angle C12 51 C13 51 C14 51 HARMONIC 1.9378399004 0.2

Angle C13 51 C14 51 C15 51 HARMONIC 2.09674861599 0.2

Dihedral C11 51 C12 51 C13 51 C14 51 CIRCULARHARMONIC 3.00264292497 0.2

Angle C11 51 C12 51 C13 51 HARMONIC 2.10142350267 0.2

Angle C12 51 C13 51 C14 51 HARMONIC 1.9378399004 0.2

Dihedral C10 51 C11 51 C12 51 C13 51 CIRCULARHARMONIC 3.07824730307 0.2

Angle C10 51 C11 51 C12 51 HARMONIC 2.12838058345 0.2

Angle C11 51 C12 51 C13 51 HARMONIC 2.10142350267 0.2

Dihedral C9 51 C10 51 C11 51 C12 51 CIRCULARHARMONIC 3.15818566909 0.2

Angle C9 51 C10 51 C11 51 HARMONIC 2.19998940194 0.2

Angle C10 51 C11 51 C12 51 HARMONIC 2.12838058345 0.2

Dihedral C8 51 C9 51 C10 51 C11 51 CIRCULARHARMONIC 3.14032007526 0.2

Angle C8 51 C9 51 C10 51 HARMONIC 2.18042140823 0.2

Angle C9 51 C10 51 C11 51 HARMONIC 2.19998940194 0.2

Dihedral C7 51 C8 51 C9 51 C10 51 CIRCULARHARMONIC 0.015281391904 0.2

Angle C7 51 C8 51 C9 51 HARMONIC 2.24709242633 0.2

Angle C8 51 C9 51 C10 51 HARMONIC 2.18042140823 0.2

Dihedral C6 51 C7 51 C8 51 C9 51 CIRCULARHARMONIC 3.12485188829 0.2

Angle C6 51 C7 51 C8 51 HARMONIC 1.77096028484 0.2

Angle C7 51 C8 51 C9 51 HARMONIC 2.24709242633 0.2

Dihedral C5 51 C6 51 C7 51 C8 51 CIRCULARHARMONIC 3.14360581988 0.2

Angle C5 51 C6 51 C7 51 HARMONIC 2.28243739272 0.2

Angle C6 51 C7 51 C8 51 HARMONIC 1.77096028484 0.2

Dihedral C4 51 C5 51 C6 51 C7 51 CIRCULARHARMONIC 3.14049828023 0.2

Angle C4 51 C5 51 C6 51 HARMONIC 2.07367504595 0.2

Angle C5 51 C6 51 C7 51 HARMONIC 2.28243739272 0.2

Dihedral C3 51 C4 51 C5 51 C6 51 CIRCULARHARMONIC 6.28252279077 0.2

Angle C3 51 C4 51 C5 51 HARMONIC 2.10415756601 0.2

Angle C4 51 C5 51 C6 51 HARMONIC 2.07367504595 0.2

Dihedral C2 51 C3 51 C4 51 C5 51 CIRCULARHARMONIC 0.0016444413877 0.2

Angle C2 51 C3 51 C4 51 HARMONIC 2.11570036489 0.2

Angle C3 51 C4 51 C5 51 HARMONIC 2.10415756601 0.2

Dihedral C1 51 C2 51 C3 51 C4 51 CIRCULARHARMONIC 6.28248053283 0.2

Angle C1 51 C2 51 C3 51 HARMONIC 2.04629909902 0.2

Angle C2 51 C3 51 C4 51 HARMONIC 2.11570036489 0.2

Dihedral N1 51 C1 51 C2 51 C3 51 CIRCULARHARMONIC 3.14090777676 0.2

Angle N1 51 C1 51 C2 51 HARMONIC 2.25345886381 0.2

Angle C1 51 C2 51 C3 51 HARMONIC 2.04629909902 0.2

Dihedral C8 51 N1 51 C1 51 C2 51 CIRCULARHARMONIC 3.13570611823 0.2

Angle C8 51 N1 51 C1 51 HARMONIC 1.94831149737 0.2

Angle N1 51 C1 51 C2 51 HARMONIC 2.25345886381 0.2

Dihedral C17 51 N1 51 C1 51 C2 51 CIRCULARHARMONIC 6.28247091397 0.2

Angle C17 51 N1 51 C1 51 HARMONIC 2.16791438223 0.2

Angle N1 51 C1 51 C2 51 HARMONIC 2.25345886381 0.2

Dihedral C17 51 N1 51 C1 51 C6 51 CIRCULARHARMONIC 3.14133570605 0.2

Angle C17 51 N1 51 C1 51 HARMONIC 2.16791438223 0.2

Angle N1 51 C1 51 C6 51 HARMONIC 1.89634371538 0.2

Dihedral C17 51 N1 51 C8 51 C7 51 CIRCULARHARMONIC 3.14324491925 0.2

Angle C17 51 N1 51 C8 51 HARMONIC 2.16694700205 0.2

Angle N1 51 C8 51 C7 51 HARMONIC 1.90154479397 0.2

Dihedral C17 51 N1 51 C8 51 C9 51 CIRCULARHARMONIC 0.0121941442831 0.2

Angle C17 51 N1 51 C8 51 HARMONIC 2.16694700205 0.2

Angle N1 51 C8 51 C9 51 HARMONIC 2.13449112581 0.2

Dihedral C9 51 C8 51 N1 51 C1 51 CIRCULARHARMONIC 3.15895554274 0.2

Angle C9 51 C8 51 N1 51 HARMONIC 2.13449112581 0.2

Angle C8 51 N1 51 C1 51 HARMONIC 1.94831149737 0.2

Dihedral C10 51 C11 51 C20 51 C21 51 CIRCULARHARMONIC 2.82116240342 0.2

Angle C10 51 C11 51 C20 51 HARMONIC 2.10006542492 0.2

Angle C11 51 C20 51 C21 51 HARMONIC 2.01061801869 0.2

Dihedral C11 51 C20 51 C21 51 C22 51 CIRCULARHARMONIC 0.87491653251 0.2

Angle C11 51 C20 51 C21 51 HARMONIC 2.01061801869 0.2

Angle C20 51 C21 51 C22 51 HARMONIC 1.91329259275 0.2

Dihedral C20 51 C21 51 C22 51 C13 51 CIRCULARHARMONIC 5.28343184488 0.2

Angle C20 51 C21 51 C22 51 HARMONIC 1.91329259275 0.2

Angle C21 51 C22 51 C13 51 HARMONIC 1.8662965236 0.2

Dihedral C21 51 C22 51 C13 51 C12 51 CIRCULARHARMONIC 0.68608154636 0.2

Angle C21 51 C22 51 C13 51 HARMONIC 1.8662965236 0.2

Angle C22 51 C13 51 C12 51 HARMONIC 2.15012219907 0.2

Dihedral C21 51 C22 51 C13 51 C14 51 CIRCULARHARMONIC 3.81667615914 0.2

Angle C21 51 C22 51 C13 51 HARMONIC 1.8662965236 0.2

Angle C22 51 C13 51 C14 51 HARMONIC 2.19517921626 0.2

Dihedral C20 51 C21 51 C22 51 C23 51 CIRCULARHARMONIC 3.06164780092 0.2

Angle C20 51 C21 51 C22 51 HARMONIC 1.91329259275 0.2

Angle C21 51 C22 51 C23 51 HARMONIC 2.05268594903 0.2

Dihedral C21 51 C22 51 C23 51 C24 51 CIRCULARHARMONIC 1.83682540895 0.2

Angle C21 51 C22 51 C23 51 HARMONIC 2.05268594903 0.2

Angle C22 51 C23 51 C24 51 HARMONIC 1.99970386711 0.2

Dihedral C22 51 C23 51 C24 51 C15 51 CIRCULARHARMONIC 0.823339191982 0.2

Angle C22 51 C23 51 C24 51 HARMONIC 1.99970386711 0.2

Angle C23 51 C24 51 C15 51 HARMONIC 1.97927193129 0.2

Dihedral C23 51 C24 51 C15 51 C14 51 CIRCULARHARMONIC 5.48342183333 0.2

Angle C23 51 C24 51 C15 51 HARMONIC 1.97927193129 0.2

Angle C24 51 C15 51 C14 51 HARMONIC 2.00601529171 0.2

Dihedral C23 51 C24 51 C15 51 C16 51 CIRCULARHARMONIC 2.39287042367 0.2

Angle C23 51 C24 51 C15 51 HARMONIC 1.97927193129 0.2

Angle C24 51 C15 51 C16 51 HARMONIC 2.13941445602 0.2

Dihedral C13 51 C22 51 C23 51 C24 51 CIRCULARHARMONIC 5.94378346983 0.2

Angle C13 51 C22 51 C23 51 HARMONIC 1.95921395795 0.2

Angle C22 51 C23 51 C24 51 HARMONIC 1.99970386711 0.2

Dihedral C14 51 C13 51 C22 51 C23 51 CIRCULARHARMONIC 6.09244310367 0.2

Angle C14 51 C13 51 C22 51 HARMONIC 2.19517921626 0.2

Angle C13 51 C22 51 C23 51 HARMONIC 1.95921395795 0.2

Dihedral C15 51 C14 51 C13 51 C22 51 CIRCULARHARMONIC 0.230087566662 0.2

Angle C15 51 C14 51 C13 51 HARMONIC 2.09674861599 0.2

Angle C14 51 C13 51 C22 51 HARMONIC 2.19517921626 0.2

Dihedral C11 51 C12 51 C25 51 C26 51 CIRCULARHARMONIC 0.0834481232059 0.2

Angle C11 51 C12 51 C25 51 HARMONIC 2.31669172035 0.2

Angle C12 51 C25 51 C26 51 HARMONIC 2.32958842799 0.2

Dihedral C11 51 C12 51 C25 51 C30 51 CIRCULARHARMONIC 3.25773215806 0.2

Angle C11 51 C12 51 C25 51 HARMONIC 2.31669172035 0.2

Angle C12 51 C25 51 C30 51 HARMONIC 1.87130069098 0.2

Dihedral C12 51 C25 51 C26 51 C27 51 CIRCULARHARMONIC 3.17215402011 0.2

Angle C12 51 C25 51 C26 51 HARMONIC 2.32958842799 0.2

Angle C25 51 C26 51 C27 51 HARMONIC 2.09210836547 0.2

Dihedral C25 51 C26 51 C27 51 C28 51 CIRCULARHARMONIC 0.0254112481881 0.2

Angle C25 51 C26 51 C27 51 HARMONIC 2.09210836547 0.2

Angle C26 51 C27 51 C28 51 HARMONIC 2.10536814375 0.2

Dihedral C26 51 C27 51 C28 51 C29 51 CIRCULARHARMONIC 6.27479279746 0.2

Angle C26 51 C27 51 C28 51 HARMONIC 2.10536814375 0.2

Angle C27 51 C28 51 C29 51 HARMONIC 2.09820878745 0.2

Dihedral C27 51 C28 51 C29 51 C30 51 CIRCULARHARMONIC 6.25420483131 0.2

Angle C27 51 C28 51 C29 51 HARMONIC 2.09820878745 0.2

Angle C28 51 C29 51 C30 51 HARMONIC 2.08602951683 0.2

Dihedral C12 51 C25 51 C30 51 C14 51 CIRCULARHARMONIC 0.000555729669134 0.2

Angle C12 51 C25 51 C30 51 HARMONIC 1.87130069098 0.2

Angle C25 51 C30 51 C14 51 HARMONIC 1.8793726572 0.2

Dihedral C25 51 C30 51 C14 51 C15 51 CIRCULARHARMONIC 2.93025989867 0.2

Angle C25 51 C30 51 C14 51 HARMONIC 1.8793726572 0.2

Angle C30 51 C14 51 C15 51 HARMONIC 2.30605294097 0.2

Dihedral C30 51 C14 51 C15 51 C16 51 CIRCULARHARMONIC 0.416471311435 0.2

Angle C30 51 C14 51 C15 51 HARMONIC 2.30605294097 0.2

Angle C14 51 C15 51 C16 51 HARMONIC 2.13657633459 0.2

Dihedral C24 51 C15 51 C16 51 NZ 51 CIRCULARHARMONIC 0.0459821189608 0.2

Angle C24 51 C15 51 C16 51 HARMONIC 2.13941445602 0.2

Angle C15 51 C16 51 NZ 51 HARMONIC 2.19840954415 0.2

Dihedral C23 51 C24 51 C15 51 C16 51 CIRCULARHARMONIC 2.39287042367 0.2

Angle C23 51 C24 51 C15 51 HARMONIC 1.97927193129 0.2

Angle C24 51 C15 51 C16 51 HARMONIC 2.13941445602 0.2

Dihedral C5 51 C6 51 C7 51 C18 51 CIRCULARHARMONIC 1.07877699811 0.2

Angle C5 51 C6 51 C7 51 HARMONIC 2.28243739272 0.2

Angle C6 51 C7 51 C18 51 HARMONIC 1.9219785072 0.2

Dihedral C5 51 C6 51 C7 51 C19 51 CIRCULARHARMONIC 5.20461911993 0.2

Angle C5 51 C6 51 C7 51 HARMONIC 2.28243739272 0.2

Angle C6 51 C7 51 C19 51 HARMONIC 1.9201715626 0.2

Dihedral C9 51 C8 51 C7 51 C18 51 CIRCULARHARMONIC 5.17114712437 0.2

Angle C9 51 C8 51 C7 51 HARMONIC 2.24709242633 0.2

Angle C8 51 C7 51 C18 51 HARMONIC 1.94760024648 0.2

Dihedral C9 51 C8 51 C7 51 C19 51 CIRCULARHARMONIC 1.08179114595 0.2

Angle C9 51 C8 51 C7 51 HARMONIC 2.24709242633 0.2

Angle C8 51 C7 51 C19 51 HARMONIC 1.94494433249 0.2

**Appendix G.** Command to run the example design script with the provided auxiliary files. The provided Rosetta XML design script (Appendix C) and relevant files (Appendix A,B,D,E and F) is fully functional with the most recent version of Rosetta version 3.14 (<https://downloads.rosettacommons.org/downloads/academic/>) using the command below:

rosetta_scripts.default.linuxgccrelease -parser:protocol <example.xml> -script_vars resfile=<example.resfile> dihedral_cst=<example.cst> aa_comp=<example.comp> -extra_res_fa <example.fa.params> -s <input.pdb> -beta

### **References**

(1)	[Huang, P.-S.; Oberdorfer, G.; Xu, C.; Pei, X. Y.; Nannenga, B. L.; Rogers, J. M.; DiMaio, F.; Gonen, T.; Luisi, B.; Baker, D. High Thermodynamic Stability of Parametrically Designed Helical Bundles. *Science* **2014**, *346* (6208), 481–485.](http://paperpile.com/b/GVLBeS/QKff)

(2)	[Xu, C.; Lu, P.; Gamal El-Din, T. M.; Pei, X. Y.; Johnson, M. C.; Uyeda, A.; Bick, M. J.; Xu, Q.; Jiang, D.; Bai, H.; Reggiano, G.; Hsia, Y.; Brunette, T. J.; Dou, J.; Ma, D.; Lynch, E. M.; Boyken, S. E.; Huang, P.-S.; Stewart, L.; DiMaio, F.; Kollman, J. M.; Luisi, B. F.; Matsuura, T.; Catterall, W. A.; Baker, D. Computational Design of Transmembrane Pores. *Nature* **2020**, *585* (7823), 129–134.](http://paperpile.com/b/GVLBeS/bWr2)

(3)	[Brunette, T. J.; Bick, M. J.; Hansen, J. M.; Chow, C. M.; Kollman, J. M.; Baker, D. Modular Repeat Protein Sculpting Using Rigid Helical Junctions. *Proc. Natl. Acad. Sci. U. S. A.* **2020**, *117* (16), 8870–8875.](http://paperpile.com/b/GVLBeS/mhhP)

(4)	[Renfrew, P. D.; Choi, E. J.; Bonneau, R.; Kuhlman, B. Incorporation of Noncanonical Amino Acids into Rosetta and Use in Computational Protein-Peptide Interface Design. *PLoS One* **2012**, *7* (3), e32637.](http://paperpile.com/b/GVLBeS/eq4p)

(5)	[Mikoshiba, H.; Motoki, M.; Shibata, M. Novel Oxonol Dyes and High-Sensitivity Optical Recording Media Therewith. JP Patent #2005336236.](http://paperpile.com/b/GVLBeS/dDP9)

(6)	[Bandi, V. G.; Luciano, M. P.; Saccomano, M.; Patel, N. L.; Bischof, T. S.; Lingg, J. G. P.; Tsrunchev, P. T.; Nix, M. N.; Ruehle, B.; Sanders, C.; Riffle, L.; Robinson, C. M.; Difilippantonio, S.; Kalen, J. D.; Resch-Genger, U.; Ivanic, J.; Bruns, O. T.; Schnermann, M. J. Targeted Multicolor in Vivo Imaging over 1,000 Nm Enabled by Nonamethine Cyanines. *Nat. Methods* **2022**, *19* (3), 353–358.](http://paperpile.com/b/GVLBeS/JDhFT)

(7)	[Hoover, D. M.; Lubkowski, J. DNAWorks: An Automated Method for Designing Oligonucleotides for PCR-Based Gene Synthesis. *Nucleic Acids Res.* **2002**, *30* (10), e43.](http://paperpile.com/b/GVLBeS/KGwx)

(8)	[Studier, F. W. Protein Production by Auto-Induction in High Density Shaking Cultures. *Protein Expr. Purif.* **2005**, *41* (1), 207–234.](http://paperpile.com/b/GVLBeS/ITuI)

(9)	[Zheng, L.; Baumann, U.; Reymond, J.-L. An Efficient One-Step Site-Directed and Site-Saturation Mutagenesis Protocol. *Nucleic Acids Res.* **2004**, *32* (14), e115.](http://paperpile.com/b/GVLBeS/LSQR)

(10)	[Dobson, D. E.; Mahoney, E. R.; Mach, T. P.; LeTourneau, R. J.; Schmitthenner, H. F. Pentamethine Sulfobenzoindocyanine Dyes with Low Net Charge States and High Photostability. *Photochem. Photobiol. Sci.* **2020**, *19* (1), 56–65.](http://paperpile.com/b/GVLBeS/hlsu)

(11)	[Schottelius, M.; Wurzer, A.; Wissmiller, K.; Beck, R.; Koch, M.; Gorpas, D.; Notni, J.; Buckle, T.; van Oosterom, M. N.; Steiger, K.; Ntziachristos, V.; Schwaiger, M.; van Leeuwen, F. W. B.; Wester, H.-J. Synthesis and Preclinical Characterization of the PSMA-Targeted Hybrid Tracer PSMA-I&F for Nuclear and Fluorescence Imaging of Prostate Cancer. *J. Nucl. Med.* **2019**, *60* (1), 71–78.](http://paperpile.com/b/GVLBeS/fypV)

(12)	[Kabsch, W. XDS. *Acta Crystallogr. D Biol. Crystallogr.* **2010**, *66* (Pt 2), 125–132.](http://paperpile.com/b/GVLBeS/mG2u)

(13)	[Winn, M. D.; Ballard, C. C.; Cowtan, K. D.; Dodson, E. J.; Emsley, P.; Evans, P. R.; Keegan, R. M.; Krissinel, E. B.; Leslie, A. G. W.; McCoy, A.; McNicholas, S. J.; Murshudov, G. N.; Pannu, N. S.; Potterton, E. A.; Powell, H. R.; Read, R. J.; Vagin, A.; Wilson, K. S. Overview of the CCP4 Suite and Current Developments. *Acta Crystallogr. D Biol. Crystallogr.* **2011**, *67* (Pt 4), 235–242.](http://paperpile.com/b/GVLBeS/LWbs)

(14)	[McCoy, A. J.; Grosse-Kunstleve, R. W.; Adams, P. D.; Winn, M. D.; Storoni, L. C.; Read, R. J. Phaser Crystallographic Software. *J. Appl. Crystallogr.* **2007**, *40* (Pt 4), 658–674.](http://paperpile.com/b/GVLBeS/hQgD)

(15)	[Adams, P. D.; Afonine, P. V.; Bunkóczi, G.; Chen, V. B.; Davis, I. W.; Echols, N.; Headd, J. J.; Hung, L.-W.; Kapral, G. J.; Grosse-Kunstleve, R. W.; McCoy, A. J.; Moriarty, N. W.; Oeffner, R.; Read, R. J.; Richardson, D. C.; Richardson, J. S.; Terwilliger, T. C.; Zwart, P. H. PHENIX: A Comprehensive Python-Based System for Macromolecular Structure Solution. *Acta Crystallogr. D Biol. Crystallogr.* **2010**, *66* (Pt 2), 213–221.](http://paperpile.com/b/GVLBeS/dWjo)

(16)	[Emsley, P.; Cowtan, K. Coot: Model-Building Tools for Molecular Graphics. *Acta Crystallogr. D Biol. Crystallogr.* **2004**, *60* (Pt 12 Pt 1), 2126–2132.](http://paperpile.com/b/GVLBeS/eRhN)

(17)	[Williams, C. J.; Headd, J. J.; Moriarty, N. W.; Prisant, M. G.; Videau, L. L.; Deis, L. N.; Verma, V.; Keedy, D. A.; Hintze, B. J.; Chen, V. B.; Jain, S.; Lewis, S. M.; Arendall, W. B., 3rd; Snoeyink, J.; Adams, P. D.; Lovell, S. C.; Richardson, J. S.; Richardson, D. C. MolProbity: More and Better Reference Data for Improved All-Atom Structure Validation. *Protein Sci.* **2018**, *27* (1), 293–315.](http://paperpile.com/b/GVLBeS/tFwB)

(18)	[Kessels, M. M.; Qualmann, B. Syndapins Integrate N-WASP in Receptor-Mediated Endocytosis. *EMBO J.* **2002**, *21* (22), 6083–6094.](http://paperpile.com/b/GVLBeS/WC2j)

(19)	[Fuenzalida-Werner, J. P.; Mishra, K.; Stankevych, M.; Klemm, U.; Ntziachristos, V.; Stiel, A. C. Alginate Beads as a Highly Versatile Test-Sample for Optoacoustic Imaging. *Photoacoustics* **2022**, *25* (100301), 100301.](http://paperpile.com/b/GVLBeS/zlcz)
